# Complex modeling with detailed temporal predictors does not improve health records-based suicide risk prediction

**DOI:** 10.1038/s41746-023-00772-4

**Published:** 2023-03-23

**Authors:** Susan M. Shortreed, Rod L. Walker, Eric Johnson, Robert Wellman, Maricela Cruz, Rebecca Ziebell, R. Yates Coley, Zimri S. Yaseen, Sai Dharmarajan, Robert B. Penfold, Brian K. Ahmedani, Rebecca C. Rossom, Arne Beck, Jennifer M. Boggs, Greg E. Simon

**Affiliations:** 1 Kaiser Permanente Washington Health Research Institute, 1730 Minor Avenue, Ste 1600, Seattle, WA 98101 USA; 2 Department of Biostatistics, University of Washington, 1705 NE Pacific St, Seattle, WA 98195 USA; 3 U.S. Food and Drug Administration, Silver Spring, MD USA; 4 Center for Health Policy & Health Services Research, Henry Ford Health System, 1 Ford Place, Detroit, MI 48202 USA; 5 HealthPartners Institute, Division of Research, 8170 33rd Ave S, Minneapolis, MN 55425 USA; 6 Kaiser Permanente Colorado Institute for Health Research, 2550 S. Parker Road, Suite 200, Aurora, CO 80014 USA

**Keywords:** Health care, Medical research

## Abstract

Suicide risk prediction models can identify individuals for targeted intervention. Discussions of transparency, explainability, and transportability in machine learning presume complex prediction models with many variables outperform simpler models. We compared random forest, artificial neural network, and ensemble models with 1500 temporally defined predictors to logistic regression models. Data from 25,800,888 mental health visits made by 3,081,420 individuals in 7 health systems were used to train and evaluate suicidal behavior prediction models. Model performance was compared across several measures. All models performed well (area under the receiver operating curve [AUC]: 0.794–0.858). Ensemble models performed best, but improvements over a regression model with 100 predictors were minimal (AUC improvements: 0.006–0.020). Results are consistent across performance metrics and subgroups defined by race, ethnicity, and sex. Our results suggest simpler parametric models, which are easier to implement as part of routine clinical practice, perform comparably to more complex machine learning methods.

## Introduction

Over 45,000 people died by suicide in the United States and an estimated 1.2 million people attempted suicide in 2020^1^. Reducing fatal and nonfatal self-harm is a public health priority around the globe. For clinicians, identifying patients at risk using traditional clinical risk factors is hardly better than chance^2^, and self-report questionnaires have only moderate predictive value^3,4^. For health systems, delivery of effective prevention programs will require accurate identification of risk at the population level^5,6^ For public health scientists assessing beneficial or adverse effects of therapies on suicidal behavior addressing confounding requires accurately accounting for pre-existing risk^7–9^.

Several research groups have developed and validated machine learning models to predict risk of suicide attempt and death using health records data. These risk models attempt to predict risk of suicidal behavior over follow-up periods ranging from 7 days to one year, often achieving good overall performance with area under receiving operating curves (AUCs)^10,11^ exceeding 0.80^12–22^.

These models have varied in their complexity, both in terms of the number and types of predictors included and in the modeling techniques used to estimate the models. Some final models had 10–20 predictors^12^, while others used over 2000^22^. Some groups estimated relatively simple models (e.g., penalized logistic regression), while others used more complex strategies (e.g., artificial neural networks or ensemble approaches).

Recently, complex machine learning models have been criticized as too opaque and not explainable to clinicians and patients^23^. While “black box” algorithms are one form of complexity, even relatively simple algorithms, such as logistic regression, become complex as the number and type of predictors considered increase. Many have commented on the need for trust and transparency when integrating machine learning risk prediction into clinical care^24,25^, and explainability may be necessary if practicing clinicians are to trust model-based alerts or recommendations^26^. Recent work, including case studies reporting on the racial biases that can be perpetuated through the implementation of machine learning methods^27^, has highlighted the importance of examining model performance across subgroups and provided a framework for doing so^28^.

In addition to transparency, trust, and explainability, transportability and technical ease of use can be practical barriers to implementing risk models in clinical care. As the number of predictors increases, so does the amount of information a health system must routinely access to employ a risk model in clinical care. More predictors also require more programming, and more complex models demand greater computational resources to update clinical risk scores. Discussion of complexity in clinical risk modeling often presumes a trade-off between prediction accuracy and transportability, explainability, and transparency^20,24,29^, but this may not be accurate.

Reported comparisons of simpler versus more complex models for prediction of suicidal behavior given a common set of predictors do not consistently show that more complex (and less transparent) methods improve accuracy. While some studies found that more complex models had superior performance, the gains were not always large^16,18–22,30^. For example, in a large study of 500,000 visits among 125,000 patients, Chen and colleagues^18^ found that the best performing models were ensemble models that included artificial neural networks and gradient boosting models using 425 predictors; the AUC for the top-performing ensemble model was 0.875, while the AUC for a logistic regression model with a subset of 100 predictors was 0.872. Complex models require more resources to estimate, validate, and implement, and these additional requirements must be weighed along with their practical improvements over simpler models.

In this study, we compare a well-performing previously developed logistic regression model predicting suicidal behavior following an outpatient mental health visit^15^, which uses relatively simple temporal predictors extracted from clinical data, to newly developed models that used over 1400 predictors including information about the timing, frequency, and rate of diagnoses, prescription fills, utilization, and mental health assessments in the 60 months prior to the visit. We examine if these detailed temporal predictors improve performance over the previously developed model when predictors were used in a random forest, artificial neural network, or a logistic regression (with lasso for variable selection). We evaluate if non-parametric approaches (random forest and artificial neural networks), which are able to model complex interactions and non-linear relationships, using detailed temporal predictors further improve upon logistic regression. Because detailed temporal predictors (e.g., recent *versus* distal previous suicide attempt) might offer the most advantage when predicting risk in close temporal proximity to the visit in question, we compare performance for models predicting suicidal behavior in the 30 days and 90 days following a visit. We compare performance using a variety of performance metrics and investigate performance across subgroups defined by race, ethnicity, and sex.

## Results

### Study population

Our sample for estimating and validating suicide attempt (fatal and nonfatal) risk prediction models contained 15,249,031 (59%) mental health specialty visits made by 1,507,684 people and another 10,551,857 general medical visits made by 2,592,332 (84%) people. The sample used for suicide death prediction models contained 13,981,418 (59%) mental health specialty visits made by 1,433,584 people and 9,714,817 (41%) general medical visits made by 2,470,576 people.

Table 1 describes our sample, both training and validation datasets, of mental health specialty visits and general medical visits used to estimate and validate suicide attempt models; Supplementary Table 1 describes the samples used for suicide death prediction models. Mental health specialty and general medical visit samples looked similar except for higher rates of mental health conditions among mental health specialty visits, including depression (73.1% mental health training versus 56.5% general medical, Table 1, training data) and psychotic disorders (7.9% mental health training versus 4.8% general medical, Table 1, training data). The 30- and 90-day suicide attempt rate was 0.27% and 0.65% for mental health specialty visits and 0.15% and 0.33% for general medical visits. The 90-day suicide death rate was 0.023% for mental health specialty visits and 0.014% for general medical visits. Proportions of visits followed by a suicide attempt or death overall, across racial and ethnic subgroups, and by sex are reported in Table 2.

**Table 1:** Cohort description of training and validation sample for mental health specialty visits and general medical visits used to estimate suicide attempt (fatal and nonfatal) risk prediction model.

	Mental health specialty visits				General medical visits			
Characteristic	Training		Validation		Training		Validation	
	*N*	%	*N*	%	*N*	%	*N*	%
Visits	10,674,110		4,574,921		7,399,746		3,152,111	
People	1,055,333		452,351		1,814,630		777,702	
Suicide attempt within 90 days of visit	68,179	0.64	29,910	0.65	24,205	0.33	10,559	0.33
Female	6,809,585	63.8	2,917,535	63.8	4,658,689	63.0	1,981,603	62.9
Age group (year)								
11–17	1,203,318	11.3	513,324	11.2	466,630	6.3	201,697	6.4
18–29	1,757,049	16.5	746,370	16.3	910,037	12.3	393,005	12.5
30–44	2,675,777	25.1	1,152,465	25.2	1,436,281	19.4	613,336	19.5
45–64	3,758,387	35.2	1,620,762	35.4	2,571,295	34.7	1,085,343	34.4
65 or older	1,279,579	12.0	542,000	11.8	2,015,503	27.2	858,730	27.2
Race and Ethnicity^a^								
Asian	575,790	5.4	241,079	5.3	377,044	5.1	163,118	5.2
American Indian/Alaska Native	105,359	1.0	43,814	1.0	83,460	1.1	37,278	1.2
Black/African American	937,826	8.8	400,089	8.7	586,268	7.9	251,759	8.0
Native Hawaiian/Pacific Islander	112,581	1.1	48,880	1.1	66,571	0.9	29,064	0.9
White, non-Hispanic	6,130,341	57.4	2,630,268	57.5	4,521,388	61.1	1,918,078	60.9
Hispanic ethnicity	2,589,816	24.3	1,120,187	24.5	1,600,730	21.6	682,587	21.7
Not recorded (i.e., race & ethnicity unknown)	356,691	3.3	147,921	3.2	220,416	3.0	93,637	3.0
Insurance Type								
Commercial group	7,880,707	73.8	3,380,597	73.9	4,519,739	61.1	1,918,158	60.9
High deductible	897,801	8.4	386,281	8.4	485,655	6.6	208,293	6.6
Individual coverage	1,935,265	18.1	832,699	18.2	1,661,780	22.5	705,697	22.4
Medicaid	765,952	7.2	325,685	7.1	683,477	9.2	294,736	9.4
Medicare	1,782,532	16.7	755,991	16.5	2,265,968	30.6	969,796	30.8
PHQ item 9 recorded at index visit	1,686,941	15.8	722,096	15.8	647,512	8.8	277,343	8.8
Response: 0	1,271,675	11.9	543,740	11.9	518,510	7.0	221,658	7.0
Response: 1	269,851	2.5	115,745	2.5	83,323	1.1	36,185	1.1
Response: 2	85,849	0.8	36,713	0.8	27,865	0.4	12,057	0.4
Response: 3	59,566	0.6	25,898	0.6	17,814	0.2	7,443	0.2
PHQ first 8 items recorded at index visit	1,588,334	14.9	680,686	14.9	650,013	8.8	278,422	8.8
Response: 0–4	339,846	3.2	146,334	3.2	145,933	2.0	62,569	2.0
Response: 5–10	542,376	5.1	233,253	5.1	197,128	2.7	84,534	2.7
Response: 11–15	344,902	3.2	147,926	3.2	152,240	2.1	65,270	2.1
Response: 16–20	238,242	2.2	101,691	2.2	109,449	1.5	46,896	1.5
Response: 21 or higher	122,968	1.2	51,482	1.1	45,263	0.6	19,153	0.6
Anxiety^b^	7,624,535	71.4	3,269,179	71.5	3,950,506	53.4	1,689,565	53.6
Bipolar^b^	1,392,701	13	59,8493	13.1	402,747	5.4	169,089	5.4
Depression^b^	7,801,374	73.1	3,346,230	73.1	4,180,781	56.5	1,786,933	56.7
Personality disorder^b^	1,936,921	18.1	831,921	18.2	796,739	10.8	341,844	10.8
Schizophrenia or other psychosis disorder^b^	841,916	7.9	357,430	7.8	358,643	4.8	150,803	4.8
Traumatic brain injury^b^	367,608	3.4	154,871	3.4	258,996	3.5	116,565	3.7
Prior mental health inpatient stay^c^	2,524,909	23.7	1,074,142	23.5	1,552,680	21.0	670,192	21.3
Prior mental health emergency department visit^c^	3,663,903	34.3	1,577,142	34.5	2,257,382	30.5	964,169	30.6
Prior mental health outpatient visit^b^	9,783,618	91.7	4,193,770	91.7	3,374,977	45.6	1,449,018	46.0
Prior antidepressant fill^c^	7,280,659	68.2	3,129,478	68.4	4,411,861	59.6	1,883,486	59.8
Prior benzodiazepine fill^c^	4,910,042	46.0	2,100,434	45.9	2,976,874	40.2	1,273,370	40.4
Prior first generation antipsychotic fill^c^	690,501	6.5	301,831	6.6	411,853	5.6	173,280	5.5
Prior lithium fill^c^	440,354	4.1	188,538	4.1	96,506	1.3	39,892	1.3
Prior second generation antipsychotic fill^c^	2,277,230	21.3	980,716	21.4	692,907	9.4	299,019	9.5

^a^Individuals who reported more than one listed race and ethnicity contribute to all selected racial and ethnic subgroups.

^b^At least one diagnosis in the last 60 months.

^c^At least one prescription filled in the last 60 months.

**Table 2:** Percentage of visits followed by a suicide attempt and death in validation sample for mental health specialty visits and general medical visits, overall and across racial and ethnic subgroups and sex.

	Mental health specialty visits			General medical visits		
	30-day suicide attempt rate	90-day suicide attempt rate	90-day suicide death rate	30-day suicide attempt rate	90-day suicide attempt rate	90-day suicide death rate
Overall	0.27	0.65	0.023	0.15	0.33	0.014
Sex						
Male	0.23	0.55	0.036	0.16	0.34	0.025
Female	0.30	0.71	0.015	0.14	0.33	0.007
Race						
American Indian/Alaska Native	0.32	0.80	0.016	0.28	0.63	0.010
Asian	0.25	0.56	0.020	0.13	0.26	0.014
Black/African American	0.22	0.52	0.002	0.15	0.33	0.006
Native Hawaiian/Pacific Islander	0.25	0.61	0.016	0.16	0.33	0.004
White, non-Hispanic	0.29	0.71	0.027	0.16	0.36	0.016
Hispanic ethnicity	0.26	0.62	0.014	0.11	0.27	0.006

### Overall performance for suicide attempt models

**Fig. 1:**
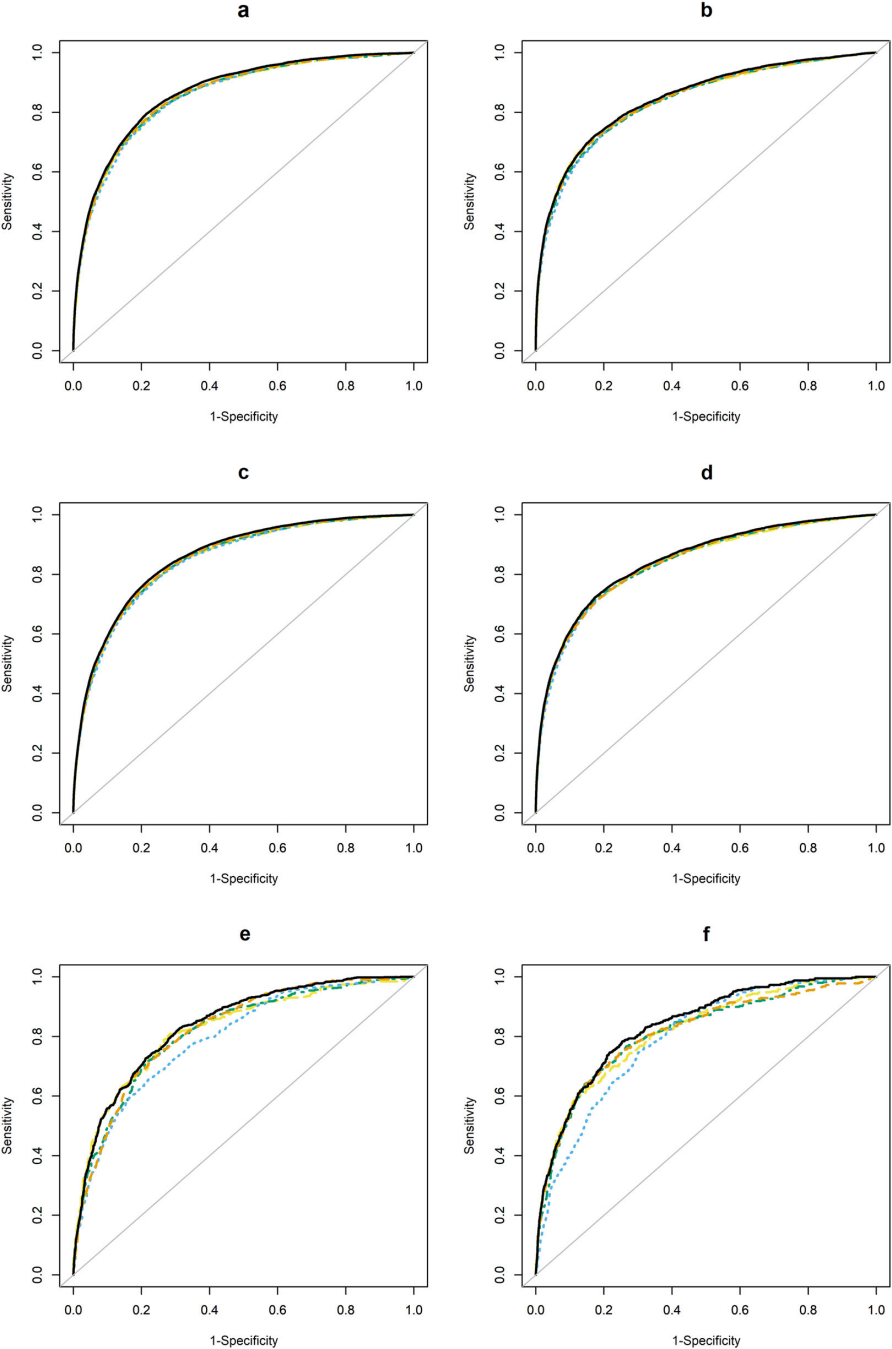
Receiver operating characteristic (ROC) curves for each prediction modeling approach (using optimal tuning parameters, estimated in the validation dataset) in both settings (mental health specialty and general medical) for 30- and 90-day suicide attempt (fatal and nonfatal) models and 90-day suicide death models. Each figure panel represents ROC curves for different samples, outcomes, and follow-up periods. (**a**): 30-day suicide attempt prediction models, mental health specialty visits; (**b**): 30-day suicide attempt prediction models, general medical visits; (**c**): 90-day suicide attempt prediction models, mental health specialty visits; (**d**): 90-day suicide attempt prediction models, general medical visits; (**e**): 90-day suicide prediction models, mental health specialty visits; (**f**): 30-day suicide prediction models, general medical visits. Original parsimonious (yellow long-dashed line); Logistic regression with lasso variable selection (green long-short-dashed line); Random forest (blue short-dashed line); Artificial neural network (orange medium-dashed line); Ensemble = ensemble model using logistic regression, random forests, and artificial neural networks with detailed temporal predictors (solid black line).

**Fig. 2:**
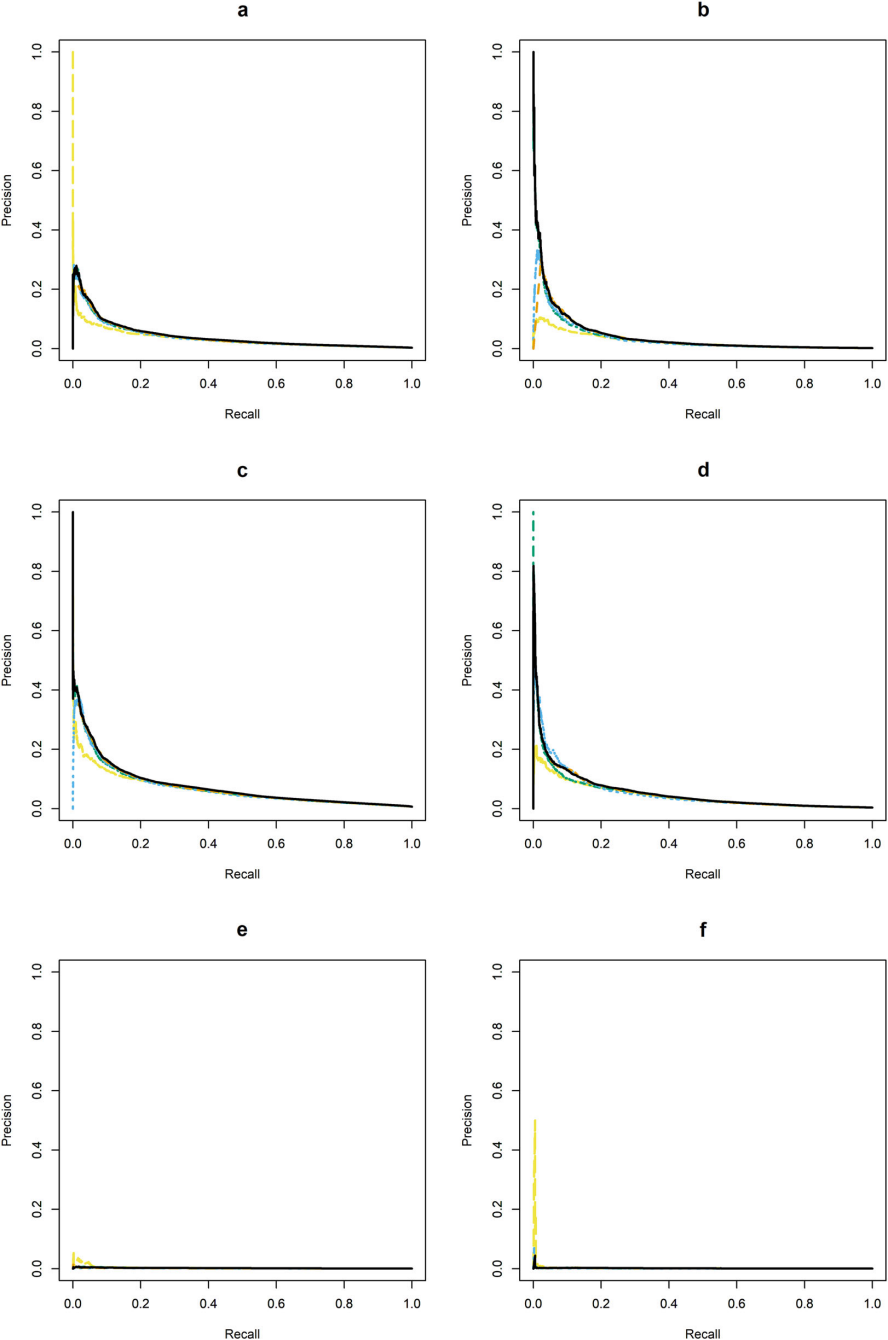
Precision-recall curves for each prediction modeling approach (using optimal tuning parameters, estimated in the validation dataset) in in both settings (mental health specialty and general medical) for 30- and 90-day suicide attempt (fatal and nonfatal) models and 90-day suicide death models. Each figure panel represents precision-recall curves for different samples, outcomes, and follow-up periods. (**a**): 30-day suicide attempt prediction models, mental health specialty visits; (**b**): 30-day suicide attempt prediction models, general medical visits; (**c**): 90-day suicide attempt prediction models, mental health specialty visits; (**d**): 90-day suicide attempt prediction models, general medical visits; (**e**): 90-day suicide prediction models, mental health specialty visits, (**f**): 30-day suicide prediction models, general medical visits. Original parsimonious (yellow long-dashed line); Logistic regression with lasso variable selection (green long-short-dashed line); Random forest (blue short-dashed line); Artificial neural network (orange medium-dashed line); Ensemble = ensemble model using logistic regresssion, random forest, and artificial neural network models with detailed temporal predictors (solid black line).

The performance, estimated in the validation sample, of each modeling strategy predicting suicide attempt in the 90 days following an index visit in the mental health specialty and general medical samples are presented in Table 3. (For information on tuning parameter selection, see Supplementary Tables 3–8). The best performing model for 90-day suicide attempts in the mental health specialty sample was the ensemble model using all three models and detailed temporal predictors (referred to as: full ensemble model) with an AUC of 0.858 (95% confidence interval: 0.856, 0.860). However, the AUCs for all suicide attempt models in this sample were very similar, including the original parsimonious model with fewer, less rich temporal predictors (AUCs ranging from 0.846 to 0.858). Using the 99th percentile as a cutoff yielded a sensitivity of 0.182 (0.178, 0.186) and a PPV of 0.111 (0.109, 0.114) for the full ensemble model compared to a sensitivity of 0.160 (0.156, 0.164) and a PPV of 0.104 (0.102, 0.107) for the original parsimonious model. The F-score showed similar patterns, with the ensemble model having a value of 0.138 (0.135, 0.141), and the original parsimonious model having a value of 0.126 (0.123, 0.130). Plots of receiver operating characteristic (ROC) curves (Fig. 1) show very little variability among models. While there is some variation across models in precision-recall curves (Fig. 2) at lower recall rates (i.e., sensitivity), this portion of the graph represents less than 1% of visits. As seen in the calibration results in Table 4, all models are well-calibrated throughout the risk score distribution, with two exceptions: the original parsimonious model and logistic regression model with detailed temporal predictors both over-estimate the probability of a suicide attempt in the highest risk group. Performance of the 90-day suicide attempt model for general medical visits was slightly lower across all metrics, yet similar patterns as the mental health specialty visits were observed; the full ensemble model had the best performance (AUC: 0.847 [0.842, 0.851]), yet all models performed similarly (AUCs ranging from 0.839 to 0.847, Table 3). Performance of 30-day suicide attempt models was similar to 90-day models for both samples (Table 5); while ensemble models using detailed temporal predictors performed the best (mental health specialty AUC 0.867 [0.864, 0.870]; general medical 0.848 [0.842, 0.854]), the improvement over the original parsimonious model was small (mental health specialty AUC 0.857 [0.853, 0.860]; general medical 0.842 [0.836, 0.849]).

**Table 3:** Prediction performance in entire validation data for suicide attempt in the 90 days following an outpatient visit; 95% confidence intervals (CIs) constructed using 10,000 bootstrap samples.

Prediction Model	AUC (95% CI)	Brier score (95% CI)	F-score of 99th percentile (95% CI)	Sensitivity of 99th percentile (95% CI)	Specificity of 99th percentile (95% CI)	PPV^†^ of 99th percentile (95% CI)
90-day suicide attempt following mental health specialty visits.						
OP	0.848 (0.846, 0.851)	6.4 × 10^−3^ (6.3,6.4) x10^−3^	0.126 (0.123, 0.130)	0.160 (0.156, 0.164)	0.991 (0.991, 0.991)	0.104 (0.102, 0.107)
LR	0.850 (0.848, 0.853)	6.4 × 10^−3^ (6.3,6.4) x10^−3^	0.132 (0.129, 0.135)	0.181 (0.176, 0.185)	0.990 (0.990, 0.990)	0.104 (0.101, 0.106)
RF	0.846 (0.844, 0.849)	6.3 × 10^−3^ (6.2,6.4) x10^−3^	0.135 (0.131, 0.138)	0.172 (0.167, 0.176)	0.991 (0.991, 0.991)	0.111 (0.108, 0.114)
ANN	0.853 (0.850, 0.855)	6.3 × 10^−3^ (6.2,6.3) x10^−3^	0.136 (0.133, 0.140)	0.172 (0.167, 0.176)	0.991 (0.991, 0.991)	0.113 (0.110, 0.116)
Ensemble: LR/RF	0.857 (0.855, 0.859)	6.3 × 10^−3^ (6.2,6.3) x10^−3^	0.137 (0.134, 0.140)	0.183 (0.178, 0.187)	0.990 (0.990, 0.990)	0.110 (0.107, 0.112)
Ensemble: RF/ANN	0.857 (0.855, 0.859)	6.3 × 10^−3^ (6.2,6.3) x10^−3^	0.138 (0.135, 0.141)	0.174 (0.170, 0.179)	0.991 (0.991, 0.991)	0.114 (0.111, 0.117)
Ensemble: LR/ANN	0.854 (0.852, 0.856)	6.3 × 10^−3^ (6.2,6.4) x10^−3^	0.135 (0.132,0.139)	0.180 (0.176, 0.184)	0.990 (0.990, 0.990)	0.108 (0.106, 0.111)
Ensemble: LR/RF/ANN	0.858 (0.856, 0.860)	6.3 × 10^−3^ (6.2,6.3) x10^−3^	0.138 (0.135, 0.141)	0.182 (0.178, 0.186)	0.990 (0.990, 0.991)	0.111 (0.109, 0.114)
90-day suicide attempt following mental health visits to a general medical provider.						
OP	0.839 (0.834, 0.843)	3.3 × 10^−3^ (3.2,3.3) x10^−3^	0.105 (0.101, 0.109)	0.214 (0.206, 0.222)	0.990 (0.990, 0.991)	0.070 (0.067, 0.072)
LR	0.839 (0.835, 0.843)	3.2 × 10^−3^ (3.2,3.2) x10^−3^	0.103 (0.099, 0.107)	0.211 (0.203, 0.219)	0.990 (0.990, 0.990)	0.068 (0.066, 0.071)
RF	0.840 (0.836, 0.844)	3.3 × 10^−3^ (3.2,3.3) x10^−3^	0.099 (0.095, 0.103)	0.211 (0.203, 0.218)	0.990 (0.990, 0.990)	0.065 (0.062, 0.067)
ANN	0.840 (0.836, 0.844)	3.3 × 10^−3^ (3.2,3.3) x10^−3^	0.109 (0.105, 0.114)	0.222 (0.214, 0.230)	0.990 (0.990, 0.991)	0.073 (0.070, 0.075)
Ensemble: LR/RF	0.846 (0.842, 0.850)	3.3 × 10^−3^ (3.2,3.3) x10^−3^	0.106 (0.102, 0.110)	0.221 (0.213, 0.229)	0.990 (0.990, 0.990)	0.070 (0.067, 0.073)
Ensemble: RF/ANN	0.846 (0.842, 0.850)	3.3 × 10^−3^ (3.2,3.3) x10^−3^	0.110 (0.106, 0.114)	0.230 (0.222, 0.239)	0.990 (0.990, 0.990)	0.072 (0.069, 0.075)
Ensemble: LR/ANN	0.841 (0.837, 0.846)	3.3 × 10^−3^ (3.2,3.3) x10^−3^	0.107 (0.103, 0.111)	0.221 (0.212, 0.229)	0.990 (0.990, 0.990)	0.071 (0.068, 0.074)
Ensemble: LR/RF/ANN	0.847 (0.842, 0.851)	3.3 × 10^−3^ (3.2,3.3) x10^−3^	0.109 (0.105, 0.113)	0.224 (0.216, 0.232)	0.990 (0.990, 0.990)	0.072 (0.069, 0.074)

*AUC* area under the receiver operating curve, *PPV* positive predicted value, *OP* original parsimonious, *LR* logistic regression with Lasso variable selection, *RF* random forest, *ANN* artificial neural network.

**Table 4:** Calibration tables for all models in mental health specialty and general medical samples with percentiles defined on training dataset and applied to validation dataset.

	OP		LR		RF		ANN		Full ensemble	
Percentile	Avg $\hat p$	Obs rate	Avg $\hat p$	Obs rate	Avg $\hat p$	Obs rate	Avg $\hat p$	Obs rate	Avg $\hat p$	Obs rate
30-day suicide attempt (fatal and nonfatal) prediction models, mental health specialty										
0–50%	0.05	0.04	0.05	0.04	0.05	0.04	0.04	0.04	0.05	0.03
50–75%	0.15	0.13	0.14	0.12	0.16	0.14	0.15	0.13	0.15	0.12
75–90%	0.34	0.38	0.31	0.34	0.37	0.39	0.38	0.38	0.35	0.37
90–95%	0.73	0.80	0.65	0.71	0.74	0.71	0.82	0.84	0.73	0.74
95–99%	1.93	1.84	1.57	1.69	1.69	1.66	1.65	1.68	1.61	1.72
99–100%	7.69	4.99	8.78	4.95	5.19	5.32	5.87	5.62	6.41	5.49
30-day suicide attempt (fatal and nonfatal) prediction models, general medical										
0–50%	0.03	0.03	0.03	0.03	0.03	0.03	0.03	0.03	0.03	0.03
50–75%	0.07	0.07	0.08	0.07	0.09	0.07	0.09	0.07	0.09	0.07
75–90%	0.16	0.15	0.16	0.15	0.20	0.18	0.21	0.15	0.19	0.16
90–95%	0.35	0.41	0.31	0.33	0.37	0.39	0.42	0.37	0.37	0.36
95–99%	0.87	0.90	0.72	0.75	0.74	0.76	0.99	0.83	0.82	0.82
99–100%	4.62	3.43	4.81	3.28	3.28	3.30	4.59	3.75	4.05	3.53
90-day suicide attempt (fatal and nonfatal) prediction models, mental health specialty										
0–50%	0.13	0.10	0.13	0.10	0.11	0.11	0.07	0.09	0.12	0.09
50–75%	0.36	0.35	0.35	0.32	0.39	0.34	0.28	0.36	0.35	0.33
75–90%	0.84	0.95	0.76	0.84	0.89	0.93	0.74	0.93	0.80	0.92
90–95%	1.82	1.96	1.58	1.93	1.75	1.86	1.60	1.96	1.64	1.77
95–99%	4.56	4.27	3.74	3.98	3.83	3.90	3.22	4.22	3.53	4.25
99–100%	14.94	10.44	16.02	10.37	10.91	11.09	11.58	11.29	12.30	11.13
90-day suicide attempt (fatal and nonfatal) prediction models, general medical										
0–50%	0.07	0.07	0.07	0.07	0.07	0.06	0.07	0.06	0.08	0.06
50–75%	0.16	0.16	0.18	0.17	0.20	0.17	0.15	0.15	0.18	0.16
75–90%	0.36	0.37	0.37	0.38	0.44	0.42	0.32	0.36	0.38	0.38
90–95%	0.79	0.97	0.73	0.89	0.85	0.90	0.66	0.82	0.75	0.86
95–99%	1.92	2.09	1.66	2.13	1.73	1.93	1.56	2.17	1.63	2.13
99–100%	8.56	6.96	8.83	6.85	6.67	6.49	8.29	7.26	7.57	7.17
90-day suicide death prediction models, mental health specialty										
0–50%	0.00	0.01	0.00	0.00	0.00	0.01	0.00	0.00	0.00	0.00
50–75%	0.01	0.01	0.01	0.01	0.02	0.02	0.01	0.02	0.01	0.02
75–90%	0.03	0.03	0.03	0.04	0.04	0.03	0.02	0.04	0.03	0.03
90–95%	0.06	0.07	0.06	0.07	0.07	0.07	0.05	0.07	0.06	0.08
95–99%	0.15	0.14	0.15	0.13	0.12	0.12	0.14	0.14	0.13	0.15
99–100%	0.64	0.35	0.71	0.28	0.25	0.25	0.70	0.21	0.47	0.30
90-day suicide death prediction models, general medical										
0–50%	0.00	0.00	0.00	0.00	0.00	0.00	0.00	0.00	0.00	0.00
50–75%	0.01	0.01	0.01	0.01	0.01	0.01	0.01	0.01	0.01	0.01
75–90%	0.02	0.01	0.02	0.02	0.03	0.02	0.03	0.02	0.03	0.02
90–95%	0.05	0.04	0.04	0.04	0.04	0.03	0.07	0.04	0.05	0.04
95–99%	0.10	0.08	0.09	0.08	0.08	0.08	0.17	0.08	0.10	0.07
99–100%	0.43	0.23	0.71	0.20	0.19	0.11	0.96	0.21	0.56	0.25

*OP* original parsimonious, *LR* logistic regression with lasso variable selection, *RF* random forest, *ANN* artificial neural network, *Avg* $\hat p$ the average predicted risk score in the subgroup, *Obs rate* observed outcome rate in the subgroup.

**Table 5:** Prediction performance in entire validation data for suicide attempt in the 30 days following an outpatient visit; 95% confidence intervals (CIs) constructed using 10,000 bootstrap samples.

Prediction model	AUC (95% CI)	Brier score (95% CI)	F-score of 99th percentile (95% CI)	Sensitivity of 99th percentile (95% CI)	Specificity of 99th percentile (95% CI)	PPV of 99th percentile (95% CI)
30-day suicide attempt following mental health specialty visits.						
OP	0.857 (0.853, 0.860)	2.7 × 10^−3^ (2.6,2.7) x10^−3^	0.078 (0.075, 0.082)	0.183 (0.176, 0.190)	0.990 (0.990, 0.991)	0.050 (0.048, 0.052)
LR^‡^	0.858 (0.855, 0.862)	2.7 × 10^−3^ (2.7,2.8) x10^−3^	0.081 (0.078, 0.084)	0.224 (0.217, 0.232)	0.988 (0.988, 0.988)	0.049 (0.048, 0.051)
RF	0.855 (0.853, 0.859)	2.7 × 10^−3^ (2.6,2.7) x10^−3^	0.084 (0.081, 0.087)	0.201 (0.195, 0.208)	0.990 (0.990, 0.990)	0.053 (0.051, 0.055)
ANN	0.860 (0.857, 0.863)	2.7 × 10^−3^ (2.6,2.7) x10^−3^	0.088 (0.085, 0.092)	0.207 (0.200, 0.214)	0.990 (0.990, 0.991)	0.056 (0.054, 0.058)
Ensemble: LR/RF	0.866 (0.863, 0.869)	2.7 × 10^−3^ (2.6,2.7) x10^−3^	0.087 (0.084, 0.090)	0.227 (0.220, 0.235)	0.989 (0.989, 0.989)	0.054 (0.052, 0.055)
Ensemble: RF/ANN	0.866 (0.863, 0.869)	2.7 × 10^−3^ (2.6,2.7) x10^−3^	0.089 (0.086, 0.092)	0.210 (0.203, 0.217)	0.990 (0.990, 0.991)	0.057 (0.055, 0.059)
Ensemble: LR/ANN	0.863 (0.862, 0.866)	2.7 × 10^−3^ (2.6,2.7) x10^−3^	0.086 (0.083, 0.089)	0.225 (0.218, 0.232)	0.989 (0.989, 0.989)	0.053 (0.052, 0.055)
Ensemble: LR/RF/ANN	0.867 (0.864, 0.870)	2.7 × 10^−3^ (2.6,2.7) x10^−3^	0.088 (0.085, 0.091)	0.225 (0.218, 0.232)	0.989 (0.989, 0.990)	0.055 (0.053, 0.057)
30-day suicide attempt following mental health visits to a general medical provider.						
OP	0.842 (0.836, 0.849)	1.4 × 10^−3^ (1.4,1.5) x10^−3^	0.060 (0.057, 0.063)	0.241 (0.228, 0.253)	0.990 (0.990, 0.990)	0.034 (0.032, 0.036)
LR	0.839 (0.832, 0.845)	1.4 × 10^−3^ (1.4,1.5) x10^−3^	0.058 (0.055, 0.061)	0.263 (0.251, 0.276)	0.989 (0.988, 0.989)	0.033 (0.031, 0.035)
RF	0.838 (0.832, 0.845)	1.4 × 10^−3^ (1.4,1.5) x10^−3^	0.058 (0.055, 0.061)	0.247 (0.234, 0.259)	0.989 (0.989, 0.990)	0.033 (0.031, 0.035)
ANN	0.843 (0.836, 0.849)	1.4 × 10^−3^ (1.4,1.5) x10^−3^	0.066 (0.062, 0.069)	0.259 (0.247, 0.272)	0.990 (0.990, 0.990)	0.038 (0.035, 0.040)
Ensemble: LR/RF	0.847 (0.841, 0.853)	1.4 × 10^−3^ (1.4,1.5) x10^−3^	0.060 (0.056, 0.063)	0.258 (0.246, 0.271)	0.989 (0.989, 0.989)	0.034 (0.032, 0.036)
Ensemble: RF/ANN	0.846 (0.840, 0.852)	1.4 × 10^−3^ (1.4,1.5) x10^−3^	0.064 (0.060, 0.067)	0.261 (0.249, 0.274)	0.990 (0.990, 0.990)	0.036 (0.034, 0.038)
Ensemble: LR/ANN	0.844 (0.838, 0.851)	1.4 × 10^−3^ (1.4,1.5) x10^−3^	0.063 (0.060, 0.066)	0.265 (0.252, 0.278)	0.989 (0.989, 0.990)	0.036 (0.034, 0.038)
Ensemble: LR/RF/ANN	0.848 (0.842, 0.854)	1.4 × 10^−3^ (1.4,1.5) x10^−3^	0.062 (0.059, 0.066)	0.262 (0.249, 0.275)	0.990 (0.989, 0.990)	0.035 (0.033, 0.037)

*AUC* area under the receiver operating curve, *PPV* positive predicted value, *OP* original parsimonious, *LR* logistic regression with lasso variable selection, *RF* random forest, *ANN* artificial neural network.

### Overall performance for suicide death models

Performance of prediction models estimating suicide death within 90 days of a mental health specialty visit was more variable than suicide attempt models (Table 6, AUCs 0.794–0.837 for mental health specialty and 0.794–0.836 for general medical). Larger performance gains were observed for the 90-day suicide death full ensemble model (mental health specialty AUC 0.837 [0.825, 0.849]; general medical 0.836 [0.816, 0.854]) over the original parsimonious model (mental health specialty AUC 0.823 [0.808, 0.837]; general medical 0.816 [0.794, 0.837]) than were seen for the 90-day suicide attempt model. Assessing sensitivity at the 99th percentile in the mental health specialty sample showed that, while the ensemble model made up of logistic regression and an artificial neural network had a larger point estimate (0.139 [0.118, 0.161]) than the full ensemble model (0.136 [0.115, 0.157]), confidence intervals overlapped to a large degree. The full ensemble model was the top performing model in the general medical sample across all performance measures.

**Table 6:** Prediction performance in entire validation data for suicide death in the 90 days following an outpatient visit; 95% confidence intervals (CIs) constructed using 10,000 bootstrap samples.

Prediction Model	AUC (95% CI)	Brier score (95% CI)	F-score of 99th percentile (95% CI)	Sensitivity of 99th percentile (95% CI)	Specificity of 99th percentile (95% CI)	PPV of 99th percentile (95% CI)
Suicide death in the 90 days following a mental health specialty visit.						
OP	0.823 (0.808, 0.837)	2.3 × 10^−3^ (2.2,2.4) x10^−3^	0.007 (0.006, 0.008)	0.153 (0.131, 0.177)	0.990 (0.990, 0.990)	0.004 (0.003, 0.004)
LR	0.815 (0.801, 0.828)	2.3 × 10^−3^ (2.2,2.5) x10^−3^	0.006 (0.005, 0.007)	0.124 (0.105, 0.145)	0.990 (0.990, 0.990)	0.003 (0.002, 0.003)
RF	0.794 (0.780, 0.808)	2.3 × 10^−3^ (2.2,2.4) x10^−3^	0.005 (0.004, 0.006)	0.116 (0.096, 0.136)	0.989 (0.989, 0.990)	0.003 (0.002, 0.003)
ANN	0.821 (0.809, 0.833)	2.3 × 10^−3^ (2.2,2.5) x10^−3^	0.004 (0.003, 0.005)	0.092 (0.075, 0.111)	0.990 (0.990, 0.990)	0.002 (0.002, 0.002)
Ensemble: LR/RF	0.832 (0.820, 0.844)	2.3 × 10^−3^ (2.2,2.4) x10^−3^	0.005 (0.004, 0.006)	0.119 (0.100, 0.140)	0.990 (0.989, 0.990)	0.003 (0.002, 0.003)
Ensemble: RF/ANN	0.823 (0.811, 0.835)	2.3 × 10^−3^ (2.2,2.4) x10^−3^	0.005 (0.004, 0.006)	0.112 (0.093, 0.132)	0.989 (0.989, 0.989)	0.002 (0.002, 0.003)
Ensemble: LR/ANN	0.828 (0.815, 0.840)	2.3 × 10^−3^ (2.2,2.4) x10^−3^	0.006 (0.005, 0.007)	0.139 (0.118, 0.161)	0.990 (0.990, 0.990)	0.003 (0.003, 0.004)
Ensemble: LR/RF/ANN	0.837 (0.825, 0.849)	2.3 × 10^−3^ (2.2,2.4) x10^−3^	0.006 (0.005, 0.007)	0.136 (0.115, 0.157)	0.990 (0.989, 0.990)	0.003 (0.003, 0.004)
Suicide death in the 90 days following a mental health visit to a general medical provider.						
OP	0.816 (0.794, 0.837)	1.4 × 10^−3^ (1.3,1.5) x10^−3^	0.005 (0.004, 0.006)	0.170 (0.134, 0.207)	0.990 (0.990, 0.990)	0.002 (0.002, 0.003)
LR	0.812 (0.789, 0.834)	1.4 × 10^−3^ (1.3,1.6) x10^−3^	0.004 (0.003, 0.005)	0.152 (0.118, 0.188)	0.989 (0.989, 0.989)	0.002 (0.001, 0.002)
RF	0.794 (0.774, 0.813)	1.4 × 10^−3^ (1.3,1.5) x10^−3^	0.002 (0.001, 0.003)	0.088 (0.061, 0.117)	0.989 (0.989, 0.989)	0.001 (0.001, 0.001)
ANN	0.812 (0.788, 0.834)	1.4 × 10^−3^ (1.3,1.6) x10^−3^	0.004 (0.003, 0.005)	0.152 (0.118, 0.188)	0.990 (0.990, 0.990)	0.002 (0.001, 0.003)
Ensemble: LR/RF	0.827 (0.808, 0.846)	1.4 × 10^−3^ (1.3,1.5) x10^−3^	0.004 (0.003, 0.005)	0.155 (0.120, 0.191)	0.989 (0.989, 0.989)	0.002 (0.002, 0.003)
Ensemble: RF/ANN	0.833 (0.813, 0.851)	1.4 × 10^−3^ (1.3,1.5) x10^−3^	0.004 (0.003, 0.006)	0.162 (0.127, 0.199)	0.990 (0.990, 0.990)	0.002 (0.002, 0.003)
Ensemble: LR/ANN	0.824 (0.802, 0.845)	1.4 × 10^−3^ (1.3,1.6) x10^−3^	0.004 (0.003, 0.005)	0.170 (0.134, 0.207)	0.989 (0.989, 0.990)	0.002 (0.002, 0.003)
Ensemble: LR/RF/ANN	0.836 (0.816, 0.854)	1.4 × 10^-3^ (1.3,1.5) x10^-3^	0.005 (0.004, 0.006)	0.184 (0.147, 0.223)	0.990 (0.989, 0.990)	0.002 (0.002, 0.003)

*AUC* area under the receiver operating curve, *PPV* positive predicted value, *OP* original parsimonious, *LR* logistic regression with lasso variable selection, *RF* random forest, *ANN* artificial neural network.

### Model performance across demographic subgroups

**Fig. 3:**
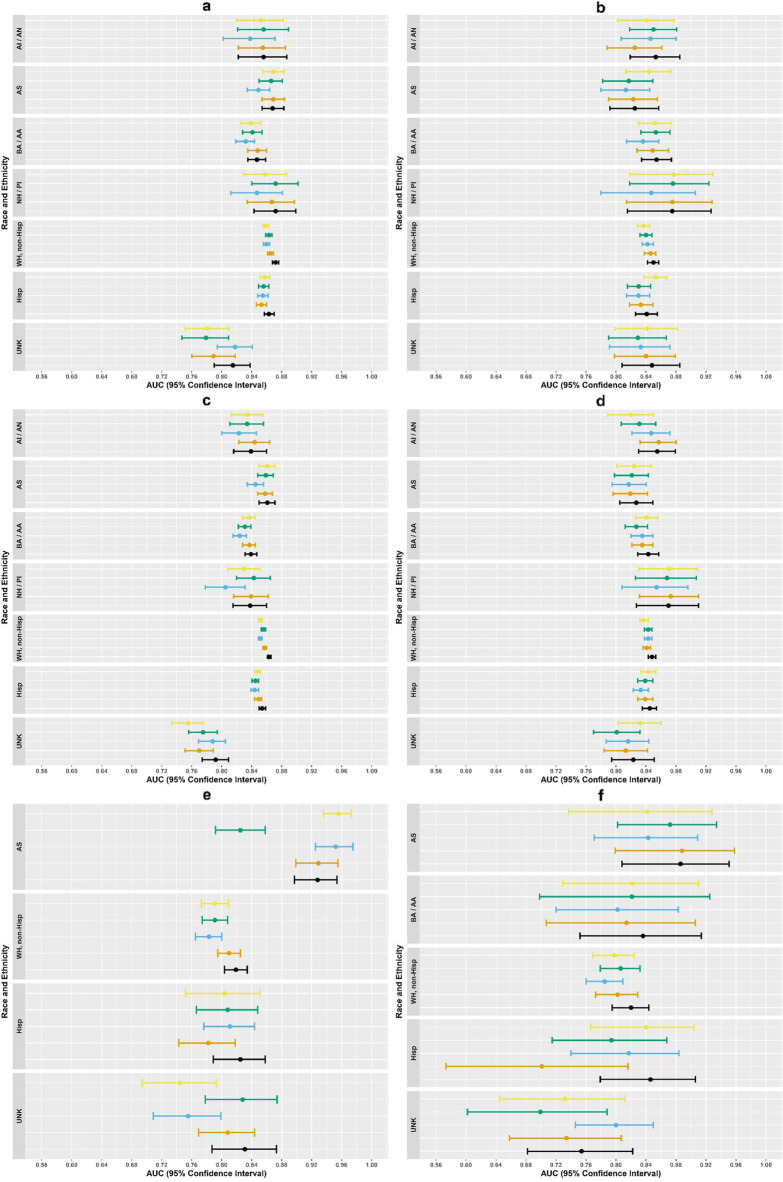
Variation in area under the receiver operating curve (AUC) across racial and ethnic groups for all suicide risk prediction models. Each figure panel represents variation in AUC across racial and ethnic groups for different samples, outcomes, and follow-up periods. (**a**): 30-day suicide attempt prediction models, mental health specialty visits; (**b**): 30-day suicide attempt prediction models, general medical visits; (**c**): 90-day suicide attempt prediction models, mental health specialty visits; (**d**): 90-day suicide attempt prediction models, general medical visits; (**e**): 90-day suicide prediction models, mental health specialty visits; (**f**): 90-day suicide prediction models, general medical visits. AI/AN = American Indian/Alaska Native; AS = Asian; BA/AA = Black/African American; NH/PI = Native Hawaiian/Pacific Islander; WH, non-Hisp = white, non-Hispanic; Hisp = Hispanic; UNK = unknown. Original parsimonious (yellow); Logistic regression with lasso variable selection (green); Random forest (blue); Artificial neural network (orange); Ensemble model using logistic regression, random forest, and artificial neural network models with detailed temporal predictors (black). Dots represent AUC in left out validation sample and lines represent upper and lower bounds on 95% confidence intervals based on 10,000 bootstrap samples. Note, due to low number of suicide deaths observed in individuals selecting AI/AN, BA/AA, or NH/PI, confidence intervals for 90-day suicide death were not constructed.

**Fig. 4:**
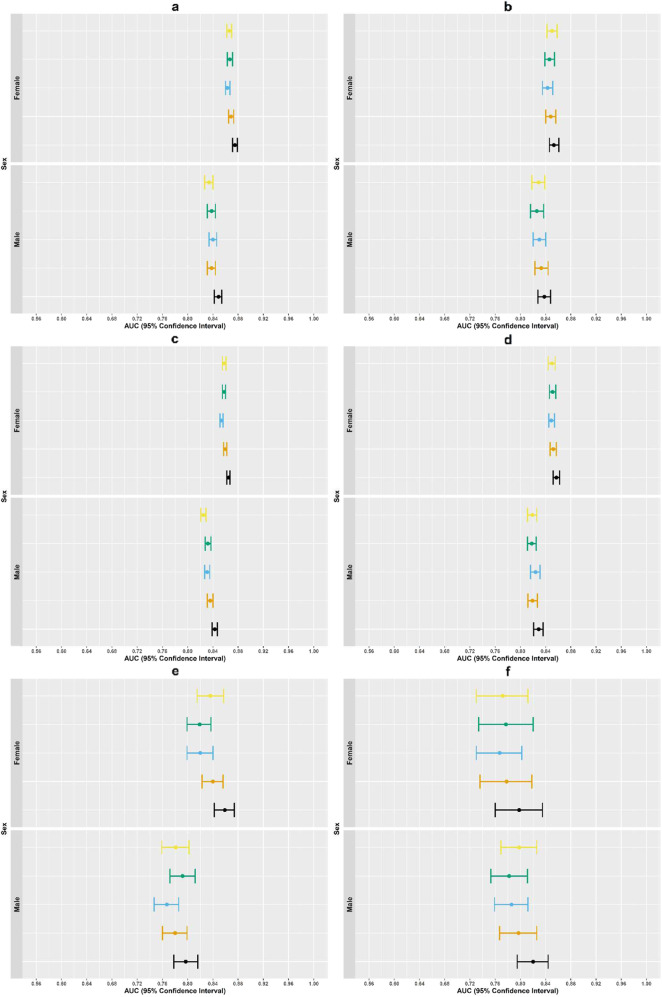
Variation in area under the receiver operating curve (AUC) across men and women for all suicide risk prediction models. Dots represent AUC in left out validation sample and the lines represent upper and lower bounds on 95% confidence intervals based on 10,000 bootstrap samples. Each figure panel represents variation in AUC across mend and women for different samples, outcomes, and follow-up periods. (**a**): 30-day suicide attempt prediction models, mental health specialty visits; (**b**): 30-day suicide attempt prediction models, general medical visits. (**c**): 90-day suicide attempt prediction models, mental health specialty visits. (**d**): 90-day suicide attempt prediction models, general medical visits. (**e**): 90-day suicide prediction models, mental health specialty visits. (**f**): 30-day suicide prediction models, general medical visits. Original parsimonious (yellow); Logistic regression with lasso variable selection (green); Random forest (blue); Artificial neural network (orange); Ensemble model using logistic regression, random forest, and artificial neural network models with detailed temporal predictors (black).

Variability in model performance across race, Hispanic ethnicity, and sex was observed, but most confidence intervals overlapped, with some confidence intervals being very wide (Figs. 3, 4, Supplementary Tables 8–12). Note, because the demographic subgroups we explored are associated with suicidal behavior and benefits of risk stratification are not present when examining performance within subgroups, we expect AUCs within subgroups to be slightly lower on average than in the full population.

Comparisons of model performance across race, ethnicity, and sex followed the same pattern as the overall sample for all outcomes and follow-up periods (30-day and 90-day suicide attempt and 90-day suicide death). The full ensemble model with detailed temporal predictors was usually among the top performers, but the best performing models provided small gains over the original parsimonious model. For example, in the mental health specialty sample for 90-day suicide attempt outcomes (Fig. 3, Supplementary Table 8), the full ensemble model had the highest AUC for all racial and ethnic subgroups (full ensemble AUCs ranged from 0.838–0.863) except for the American Indian/Alaska Native subgroups and Native Hawaiian/Pacific Islander. The random forest model had poorer performance in these subgroups (AUC 0.823 [0.800, 0.846] and 0.805 [0.778, 0.831], respectively); thus, the ensemble model with just the artificial neural network and logistic regression had the strongest performance for the Native Hawaiian/Pacific Islander subgroup (AUC 0.844 [0.821, 0.866]), and the artificial neural network model alone had the strongest performance for the American Indian/Alaska Native subgroup (AUC 0.844 [0.823, 0.864]). The AUC of the original parsimonious model across known racial and ethnic groups ranged from 0.830 to 0.861, and the differences between the best performing model and the original parsimonious model ranged from 0 to 0.011.

In the mental health specialty sample for 90-day suicide attempt, the full ensemble model with detailed temporal predictors had the highest AUC for women (0.865 [0.862, 0.867], Fig. 3, Supplementary Table 9) and men (0.843 [0.839, 0.847]); this was a modest increase over the original parsimonious models (AUC women: 0.858 [0.855, 0.861] and men: 0.825 [0.821, 0.829]).

## Discussion

In this large sample of outpatient mental health specialty visits across seven health systems, the performance of suicide risk prediction models, both parametric and non-parametric, using approximately 1,500 detailed temporal predictors was similar to previously developed parsimonious risk prediction models relying on less than 100 predictors. While a full ensemble model, averaging predictions from three models using all detailed temporal predictors, often performed best (AUCs of approximately 0.85), the improvement over the much simpler, previously developed logistic regression model was small (improvements in AUC ranging from 0.006 to 0.020; improvements in 99^th^-percentile PPV ranging from 0.000 to 0.070). This pattern held across subgroups defined by race, ethnicity, and sex, across performance metrics, and across 30- and 90-day follow-up for observing suicidal behavior.

The suicide prediction models estimated and compared in this study were developed at the visit level, which allows the models to identify both which individuals are at risk as well as when individuals are at risk. This analytic approach is in contrast to many previously developed and compared suicide risk prediction models that have relied on one observation per person, often using a case-control sampling approach, and focused on identifying who is at risk at a particular point in time given available data at that time. Most research groups have incorporated temporal information into predictors in a simplistic way, similar to the original parsimonious model examined here. In particular, Bayramli and colleagues’^21^ work, which centered on random forests and naïve Bayesian classifiers, found that including time since first visit, number of visits since the first visit, and visit rate during their time at the health system was helpful for identifying who is at high risk for suicide (AUC when no temporal variables, 0.808; AUC with temporal variables 0.824). The type of temporal information found to be important by Bayramli and colleagues^21^ has often been included in suicide risk prediction models; yet no prior work explored the importance of such detailed temporal predictors as in our study.

As discussed in the introduction, Chen and colleagues^18^ used a visit-based sampling framework similar to ours and estimated a variety of models, including random forests, logistic regression, and neural networks, to predict suicidal behavior after a mental health specialty visit using Swedish health records and national registry data. The top performing ensemble model, using all 425 predictors, provided a 0.006 improvement in AUC over a logistic model using 100 predictors. Chen and colleagues observed slightly better performance (top performing model for 90-day suicide attempt had AUC of 0.882) than observed in our study (AUC 0.858). While our models included more detailed temporal predictors, these predictors were all functions of health care services received, primarily mental health care services. In contrast, predictors used by Chen and colleagues included information on an individual’s education, employment, and known criminal offenses; parental education, employment, and known criminal offenses; and family history of disease. The magnitude of performance improvement between this model, which included social determinants and negative life events, and our model, which did not, was minimal.

Our estimated models included a large number of predictors, primarily related to mental health care utilization, including detailed and complex temporal pattern variables. Substantial effort by subject matter experts was made to curate potential predictors. This curation was likely a key contributor to the strong performance of our prediction models, including the simpler parsimonious model. This approach entails high up-front development costs but has advantages including the potential for greater face validity to clinicians and health system leaders. The relative transparency of a logistic regression can improve trust and clinician understanding of what is, and what is not, used to produce risk predictions. Transparency and trust are important for successful implementation of risk models into clinical care^31^.

Additional considerations for implementation are the personnel and computational resources required for integrating risk prediction models, with routine updating of predictions, into electronic health record systems. The US Veterans Health Administration has implemented suicide risk prediction models into clinical care and specifically elected to use a simpler model with fewer predictors^30^. Pragmatically, it is easier to write a program to create 100 predictors than it is one for 1500 predictors. Less memory is required to store logistic regression coefficients compared to complex models like random forests. Finally, basic mathematical functions (e.g., addition, multiplication, exponentiations) are fast to compute; this is important because computationally intensive prediction updates on an entire population could interfere with access to, or slow performance of, the electronic health record system, potentially compromising patient care.

Implementation of risk prediction models should consider their intended uses and the potential harms and benefits of both false positives and false negatives in those contexts. For example, suicide risk prediction models generally have low PPV. Low PPV and the potential for harm from coercive care measures (e.g., involuntary psychiatric holds) preclude reliance on risk prediction models to drive such decisions. Low-risk interventions may be more usefully informed by model-based risk stratification however even when PPV is low. Current implementations alert providers to conduct additional risk assessment^32,33^, and models that robustly concentrate risk could inform allocation of scarcer resources, e.g., therapists skilled in providing evidence-based treatments^34–36^.

Considering potential harms and benefits across all subgroups is crucial; model performance^28^ and potential for harm from intervention (e.g., “wellness” checks conducted by police) may vary by subgroup. Although estimated performance of our model did not vary by demographics, confidence intervals for some subgroups were wide. Any implementation should involve conversations with clinicians and health system leaders around these, and other limitations, of risk-modeling and appropriate clinical workflow. When more evidence-based, preventive interventions are available, these models can help target their delivery.

The strengths of our study include a large overall sample size allowing us to use large training datasets for developing prediction models while retaining a large independent validation dataset to estimate performance. The data used for model building and evaluating performance includes several million patients from broad geographic regions. We considered multiple tuning parameter values for each of the modeling strategies and used cross-validation, following recommended procedures to divide folds at the person level to protect against overfitting in our model development process^37^. We also consider the ability to identify not just who but when individuals are at high risk, inherent in our visit-based predictive modeling strategy, a strength of our work.

Our findings that detailed temporal predictors and more complex modeling strategies offered little improvement over a more parsimonious logistic regression model may be specific to this setting and these data. It is possible that including different predictors, such as information on general medical utilization, negative life events, and financial transactions, would result in meaningful differences between the methods and predictor sets we compared. Additionally, the findings we report here might not apply to different prediction targets or settings, including prediction for individuals not engaged in mental health care. Our results are also limited to the 30- and 90-day windows used for assessing suicide attempts and 90-day window for suicide deaths; it is possible that different performance and/or differences between models would be observed using different follow-up periods.

Information on gender identity and sexual orientation were not available. At the time of data extraction only sex assigned at birth was available in the health systems records data; all health systems are currently expanding collection of patients’ sexual orientation and gender identity, including gender transitions, which could improve model performance overall and among sexual and gender minorities, populations for which suicide prevention research is critical^38–40^.

Outcomes included fatal and nonfatal self-harm, certainly including some instances of self-harm without suicidal intent. Self-harm with and without suicidal intent cannot be distinguished using ICD-10-CM coding of nonfatal events or ICD-10 coding of fatal events.

Our current work compared performance of newly developed risk prediction models to performance of a previously published algorithm (original parsimonious models). We were unable to identify and exclude the visits from the current validation dataset that were used to train the previous (original parsimonious models) models; thus, it is possible that our estimates of performance of the original parsimonious model are slightly optimistic. However, there was little overfitting observed during the development of the original parsimonious models; performance in validation data was nearly identical to that in training data^15^. We also note that nonfatal suicide attempts were extracted from health records, and suicide deaths were identified from state death records. It is possible these records could misclassify suicide attempts and deaths, although recent work has observed that misclassification rates are low^41^.

The improvement in performance gained by increasing complexity of the modeling strategy and predictor set was small in this study of building risk prediction models for suicidal behavior using data from electronic health records. This improvement in performance should be considered in relation to the challenges of implementing complex models, relying on hundreds of predictors, in clinical care.

## Methods

### Study setting and population

All outpatient mental health visits made by individuals 11 years and older between January 1, 2009 and September 30, 2017 in 7 health care systems (HealthPartners, Henry Ford Health System, and the Colorado, Hawaii, Northwest, Southern California, and Washington regions of Kaiser Permanente) were included. An outpatient mental health visit was defined as an outpatient visit to a mental health specialty provider or a visit made to a general medical provider with a mental health diagnosis (referred to here as general medical visits). Predictions were made at the visit level, and people could contribute more than one visit to our sample. We use the term index visit to indicate the visits for which predictions are to be made, with predictors observed up to and including the day of the index visit and outcome information gathered after. Responsible institutional review boards for each participating health system approved waivers of consent for use of records data in this research: Henry Ford Health institutional review board ([IRB], #9998, Henry Ford Health System), Kaiser Permanente Colorado IRB (#00002931, Kaiser Permanente Colorado), and Kaiser Permanente Interregional IRB (#799744, Washington, HealthPartners, Hawaii, Northwest, and Southern California regions of Kaiser Permanente).

### Construction of detailed temporal predictors

Predictors in four categories were extracted from health records and insurance claims in the 5 years prior to the index visit: (1) demographics; (2) prior mental health diagnoses (based on ICD-9-CM and ICD-10-CM codes) and general medical diagnoses captured by the Charlson comorbidity index^42^; (3) prior mental health-related prescription fills; and (4) prior and current (i.e., on the day of the index visit) responses to the patient health questionnaire (PHQ), including both PHQ-8 total scores measuring depressive symptoms and PHQ item 9 assessing suicidal ideation^43,44^. Predictors incorporated timing, such as how many times a prior predicting event occurred in a specific time period (e.g., last 3 months or last 5 years), how recently the predicting event occurred, and how long ago the predicting event first occurred. For example, detailed temporal predictors related to depression diagnoses included: number of prior depression diagnoses in the last 5 years, number of months since most recent depression diagnosis, and number of months since first recorded depression diagnosis (within the 5 years prior to the index visit). This scheme produced 41 different temporal patterns for each category of mental health diagnosis and 23 different temporal patterns for each category of mental health medication, which resulted in 1400 detailed temporal predictors (see *Specifications for detailed temporal predictors* below for full description).

### Follow-up and outcomes

We estimated separate models for mental health specialty visits and general medical visits to predict risk of suicide attempt (both fatal and nonfatal) in the subsequent 30 and 90 days and suicide death in the subsequent 90 days; there were too few suicide deaths to estimate risk in the 30 days following a visit. In this study, suicide attempt is defined as a documented diagnosis of self-harm. Suicides were identified using state death certificates with cause of death ICD-10 mortality codes in the ranges X60-X84, Y10-Y34, Y87.0, and Y87.2. Nonfatal suicide attempts were identified using diagnosis codes from electronic health records and insurance claims. Non-fatal suicide attempts on or before September 30, 2015 were identified using ICD-9-CM diagnosis codes E950-E958 (suicide and self-inflicted injury) or E980-E988 (injury of undetermined intent). After September 30, 2015, non-fatal suicide attempts were identified using ICD-10-CM diagnosis codes were used to identify non-fatal suicide attempts. The full list of ICD-10-CM diagnosis codes used to identify non-fatal suicide attempts includes over 1000 (non-adjacent) codes and can be found at: https://github.com/MHResearchNetwork/more-srpm. We briefly summarize here. An ICD-10 era attempt was defined as either:(1) the presence of any single code from the following ranges: (a) X71-X83 (external causes of morbidity classified as intentional self-harm), (b) Y21-Y33 (external causes of morbidity of undetermined intent), (c) T36-T65 (poisoning/toxic effects) or T71 (asphyxiation) initial encounter codes with “intentional self-harm” or “undetermined intent” in the official code description, or (d) T14.91 (suicide attempt); or (2) the presence of suicidal ideation code R45.851 accompanied by an initial encounter code for a wound (S/T codes with “wound,” “laceration,” or “traumatic amputation” in the description) or poisoning/toxic effects (T codes with “poisoning” or “toxic” in the description) recorded in the same encounter.

To ensure observation of self-harm diagnoses following the index visit, analyses of suicide attempt models only included index visits for which the individual was enrolled in the health system at the index visit and for 90 days following the visit (unless an event was observed before they disenrolled; no events were excluded). We gathered outcome information through December 31, 2017; no index visit used to estimate or evaluate the suicide attempt models was censored due to study end. Visits used to develop and evaluate suicide death models were not censored for disenrollment because health systems’ research data warehouses include cause of death data from state death certificates for all current and past patients. The timing of when cause of cause of death information began and ceased to be available varied across health system. Henry Ford Health System cause of death data was available starting September 1, 2012 through December 31, 2015, Kaiser Permanente Colorado had cause of death data available from January 1, 2009 through December 31, 2017, all other health systems had cause of death data available from January 1, 2009 through December 31, 2016. Only visits with cause of death data available during the full follow-up were used to estimate and validate suicide death models.

### Training data, validation data, and tuning parameter selection

Mental health specialty and general medical visits were separately divided into independent training and validation datasets at the patient level. All visits from a randomly sampled 30% of patients were assigned to the validation dataset, and all visits from the remaining patients were defined as the training dataset; no patients contributed visits to both training and validation datasets. Division of observations into training and validation datasets was done separately for each sample (mental health specialty or general medical) and outcome (suicide attempt or suicide death).

Prediction model performance at varying combinations of tuning parameter values for each modeling method (described below) was estimated using five-fold cross validation^45,46^. Within each training dataset, a fold was defined at the person level; that is, all people in a training dataset were randomly divided into 5 folds, or groups, and all visits for an individual were included together in a fold. Due to computational burden, not all combinations of tuning parameters were considered in both samples for both outcomes. Further, additional tuning parameters were added (i.e., we widened the tuning parameter search criteria) as needed to ensure selection of parameters close to an optimum. Final tuning parameters were selected using the best out-of-fold AUC. For each outcome, setting, and modeling method, a final model was estimated on all visits in the training data using the selected tuning parameters.

### Random forest models

We constructed random forests of probability trees^47,48^. Three tuning parameters were considered: minimum node size for considering a split (1000; 10,000; 25,000; 50,000; 100,000; 250,000; 500,000 visits), number of predictors considered at each split (38, 114, 190, 380), and number of trees (10, 100, 500)^49^. The standard recommendation is to consider the square root of the number of predictors at each split, which equaled 38 for this analysis; we also considered larger numbers of predictors at each split (3, 5, and 10 times as many) to see if this improved performance^49,50^. Examining too few predictors at each split may limit tree growth if too many predictors are not associated with the outcome or are closely correlated with predictors already used for a split^49^. See Supplementary Table 2 for in-sample results across parameter settings and Supplementary Table 3 for selection of optimal tuning parameters by out-of-sample results. Random forests were estimated using R package *ranger* version 0.11.2, R version 3.5.3 (2019–03–11) and RStudio version 1.1.463.

### Artificial neural network models

We implemented feed-forward artificial neural networks (i.e., nodes in hidden layers feed information “forward” into other hidden layers, and the “last” hidden layer feeds into the final output layer)^51–53^. We used the logit (sigmoid) activation function and a small L1-penalty on the first hidden layer inputs to avoid overfitting. The number of hidden layers (1 or 2) and the number of nodes per hidden layer (4, 8, or 16) were considered tuning parameters; see Supplementary Table 4 for in-sample results and Supplementary Table 5 for selection of optimal tuning parameters. Artificial neural networks were fit using the CRAN package *Keras* version 2.2.5 with RStudio version 1.2.5001 and R version 3.6.1. We used a batch size of 2^12^ = 4,096 for the mental health specialty visits and 2^10^ = 1,024 for general medical visits, 100 epochs, and a learning rate of 0.001. Additional software needed to fit the artificial neural networks included *Tensorflow* version 2.0, *Anaconda* version 4.3.30, and *Python* version 3.6.9.

### Penalized logistic regression models

We estimated logistic regression models using lasso for variable selection and coefficient shrinkage^54^. We used a screening process to reduce the number of predictors considered in the final model while still allowing the model to consider a large number of interactions among the predictors. During this screening process we included interactions between several covariates (PHQ item 9 score recorded at index visit, sex, race, and prior suicide attempt) and most other covariates and used a small penalization term to reduce the coefficients to zero. All variables defined by interactions with non-zero coefficient values during this screening process were included along with all predictors to select the tuning parameter (lambda) and estimate the final model (see Supplementary Table 5 for in-sample results and Supplementary Table 7 for out-of-sample results). We used a grid search over 20 lambda values for each combination of outcome and sample. The same software used to implement the artificial neural networks was used to estimate the penalized logistic regression models because of the computational efficiencies and large data capacity available in the Keras package.

### Ensemble models

We estimated ensemble models by taking a simple average of predictions from all three models (logistic regression, random forest, and artificial neural networks) as well as averaging predictions from all pairs of models.

### Original parsimonious models

Performance of the above models was also compared to previously published suicide attempt and suicide death prediction models developed using outpatient mental health visits made between January 1, 2009 and June 30, 2015 in the same health systems. The sample of visits used here to develop new models includes visits used to develop these existing models. The published prediction models were logistic regression models with variables selected using lasso^15^. Predictors covered the same four categories as above but incorporated less information about the timing of predictors and fewer possible interactions: Presence/absence of a predicting event (e.g., recorded diagnosis) was measured using four different combinations of time-windows (i.e., at index visit or prior 3 months, 6 months, 1 year, and 5 years). A total of 325 predictors were considered for these models; details can be found in the online supplement of Simon et al^15^. Approximately 100 predictors were selected in the suicide attempt models, and approximately 30 predictors were selected in the suicide death models. We applied the published coefficients directly without calibration and refer to these models as the *original parsimonious models*.

### Comparing model performance on the validation sample

Final models were compared on an independent validation dataset using the following performance metrics: AUC, F-score (i.e., harmonic mean of precision and recall), and brier score (which is equivalent to the mean squared error in the binary outcome setting) as well as sensitivity, specificity, negative predictive value (NPV), and positive predictive value (PPV) at different percentile cut points. We calculated 95% confidence intervals (CIs) using the non-parametric bootstrap with 10,000 iterations. Bootstrapping was performed at the visit level to represent the variability in the population of outpatient mental health visits (rather than at the person level to represent the variability in the patient population). We present plots of receiver operating characteristic (ROC) and precision-recall curves^55^ as well as calibration tables. Performance metrics requiring cut-points (e.g., PPV, sensitivity, calibration tables) used cut-points defined in the training data to reflect the real-life situation in which deploying a model in a health care system requires pre-specifying cut-points to categorize high-risk visits.

We also calculated all performance metrics in subgroups defined by race, Hispanic ethnicity, and sex to compare model performance across subgroup categories. Self-reported race was extracted from electronic health records. This patient-reported information is usually collected at an initial primary care visit by clinic staff. Possible race categories included: American Indian/Alaska Native, Asian, Black/African American, Native Hawaiian/Pacific Islander, or white. Individuals who selected multiple races contributed to estimates of performance for all racial groups identified and individuals with missing race information were included in a group together (unknown race). Individuals could have missing information on race because they had not had a clinic visit in which this information was collected, they were not asked, or they selected to not provide this information. Hispanic ethnicity was also extracted from electronic health records and treated separately from race, except for defining a non-Hispanic white subgroup. The non-Hispanic white subgroup only included visits from individuals who selected their race as white and did not select Hispanic ethnicity. Performance in the Hispanic subgroup included all individuals reporting this ethnicity, regardless of the race they selected. Information on sex was extracted from health systems records and at the time of this data pull, most likely represents sex assigned at birth.

### Specifications for detailed temporal predictors

The unit of analysis for this work was an outpatient mental health visit or a general medical visit associated with a mental health diagnosis; we use the term “index visit” throughout to refer to the visit for which the model is being used to estimate suicide attempt risk in the following 30 or 90 days. Predictors are defined using information from the 60 months prior to the index visit. This section describes the detailed temporal predictors, that were overviewed in the main text.

Three different model types were estimated in this work: two non-parametric models (random forest and artificial neural networks) and a parametric model (logistic regression with a lasso shrinkage penalty). There are two key differences between these modeling approaches:

1. *Handling of interactions–*A strength of random forests and neural networks is their ability to find interactions to improve prediction accuracy, whereas for a logistic regression model all interactions to be considered must be specified *prior* to estimation.
2. *Handling of missing data*–In our setting, missing data occurs when the value of a predictor depends on the presence of a health care event, such as the presence of a diagnosis code. For example, possible predictors may be the number of depression diagnoses in the last year or the date of the most recent depression diagnosis; both values would be missing for someone who does not have any prior depression diagnosis. In random forest methodology missing values can be treated completely separately (i.e., a potential data split could be presence or absence of a diagnosis of depression) or be lumped with other observed values (i.e., a potential data split could be those with more than two prior depression diagnoses, with those people who have no prior depression diagnoses being grouped with those who have one prior depression diagnosis). It is not clear whether individuals with no diagnosis should be deemed “closer” to those individuals with the lowest or the highest value on the predictors scale. Thus, for many continuous-valued predictors with the potential for missing data, two predictors were created: one in which missing values were coded just below the lowest end of the scale and another in which missing values were coded slightly higher than the largest value on the scale. In parametrizing the logistic regression model, we addressed this missing data by integrating interactions into the modeling strategy, i.e., we estimated coefficients related to timing and frequency of a predictor only for those with that predicting event. For example, our model estimated a coefficient for those individuals without a depression diagnosis and then estimated a coefficient for the number of prior depression diagnoses only among those individuals with a depression diagnosis.

For these reasons (interactions and missing data), two different predictor sets were created, one for the non-parametric approaches and one for the parametric approach.

Here we outline how data gathered from the electronic health records (EHRs) of individuals was used to construct the predictors included in the analytic dataset for model building. We first provide a brief overview of the data pulled from the EHR. We then the describe analytic predictors created for estimating random forests and artificial neural networks followed by a description of the analytic predictors created for estimating penalized logistic regression models, including information about interactions considered in the screening process.

We first provide a brief summary of the data extracted from EHR for the suicide risk prediction models. Sixty monthly variables were defined for each measured type of diagnosis, health care utilization, and filled prescription (i.e., dispensing). Monthly EHR data were captured differently for diagnoses and utilization than for prescriptions. Thus, Sections A.2 and A.3 have four distinct subsections each. The first subsection is identical in both Sections and describes demographic predictors. The second subsection describes the predictors encompassing diagnoses and utilization information, the third subsection describes predictors based on filled prescriptions, and the fourth subsection describes predictors based on patient health questionnaire (PHQ) responses. Section A.3, which describes the predictor set for the logistic regression models includes a fifth subsection, which describes interactions considered. Not all patients have 60 months of health system enrollment preceding the index visit. Number of months of prior enrollment is recorded as a separate analytic predictor and months of prior enrollment is incorporated in some of predictors described below. Note, diagnoses occurring on the day of the index visit were not included in any predictors calculated using past information. When the phrase “last month” is used, it means the last month *excluding* diagnoses on the index day.

### Analytic variable (i.e., predictor) specifications for random forests and neural networks

Predictors based on demographic information:

1. Visit type (mental health [MH], general medicine).
2. Age (in years).
3. Sex (Male, Female, Unknown).
4. Race (Asian, Black or African American, Native Hawaiian/Pacific Islander, American Indian/Alaska Native, Multiple races, Other race, Unknown, white).
5. Indicator variable for Hispanic ethnicity.
6. Number of months of prior enrollment.
7. Months since first MH-related visit.
8. Indicator for if census information was available at time of visit.
9. Categorical variable for census information not available, if median household income < $25 K, if median household income ≥ $25 K but < $40 K, or ≥ $40 K.
10. Categorical variable for if census information not available, if neighborhood <25% college-educated, neighborhood ≥25% college-educated.
11. Type of insurance coverage (individual binary indicators (not necessarily mutually exclusive as individuals can have multiple coverage) for: Affordable Care Act, Medicaid, commercial, private pay (e.g., individual/family coverage), state-subsidized, self-funded, Medicare, high-deductible, other).
12. Total Charlson score and each of the Charlson subitems. Missing values set to −1, indicating a person did not have any encounters in which to observe diagnoses during 1–365 days prior to visit.

Variables summarizing 60 months of information on diagnoses and health care utilization.

We use X to denote each diagnosis or utilization type in the descriptions below. We use “X” throughout to be consistent and less repetitive, but it should always read “diagnosis or utilization type X.”

For each of the following 25 categories, the following variables were computed from EHR and insurance claims data:

- *Diagnoses* (18 in total): Depression, anxiety, bipolar, schizophrenia, other psychosis, dementia, attention deficit and hyperactivity disorder (ADHD), Autism spectrum disorder (ASD), personality disorder, alcohol use disorder, drug use disorder, post-traumatic stress disorder (PTSD), eating disorder, traumatic brain injury, conduct/disruptive disorder, diabetes, asthma, pain diagnosis.
- *Mental health-related utilization* (3 types in total): Inpatient encounters with a MH diagnosis, outpatient MH specialty encounters, emergency department encounters with MH diagnosis.
- *Prior injury*, (4 types in total): Any suicide attempt, laceration suicide attempt, other violent suicide attempt, any injury/poisoning diagnosis.

For the full list of ICD-9-CM and ICD-10-CM codes see: www.github.com/MHResearchNetwork/more-srpm

Variables summarizing total count of days with X in specific time periods:

1. Total count of days with X in last 1 month.
2. Total count of days with X in last 3 months.
3. Total count of days with X in last 12 months.
4. Total count of days with X in last 24 months.
5. Total count of days with X in last 60 months.

Variables that describe the past “rate” of X:

D06 Total days with X in last 3 months divided by number of months enrolled in those months.
D07 Total days with X in last 12 months divided by number of months enrolled in those months.
D08 Total days with X in last 24 months divided by number of months enrolled in those months.
D09 Total days with X in the past 60 months divided by number of months enrolled in those months.

Variables capturing information on how recently X occurred:

D10 Most recent occurrence of X (months prior to visit). Those who do not have X observed (ever), set value for most recent occurrence to −5 months.
D11 Most recent occurrence of X (months prior to visit). Those who do not have X observed (ever), set value for most recent occurrence to 65 months.
D12 Most recent month for which X was not observed. Those who do not have X observed (ever), set value for most recent month without X to −5.
D13 Most recent month for which X was not observed. Those who do not have X observed (ever), set value for most recent month without X to 65.

Variables describing earliest occurrence of X:

D14 Earliest occurrence of X (months prior to visit). Those who do not have X observed (ever), set value for most recent occurrence to −5 months.
D15 Earliest occurrence of X (months prior to visit). Those who do not have X observed (ever), set value for most recent occurrence to 65 months.
D16 Difference between the earliest month and most recent month with occurrence of X. Those with only 1 occurrence set difference to 0. Those who do not have X observed (ever), set difference to −5.
D17 Difference between the earliest month and most recent month with occurrence of X. Those with only 1 occurrence set difference to 0. Those who do not have X observed (ever), set difference to 65.

Variables describing trend in X over time:

D18 (# of months with X) × [(difference between the earliest month and most recent month with X] + 1). Those who do not have X observed (ever) or only have one occurrence, set value to 0.
D19 Maximum # of days with X in any month minus the minimum count of days with X in any month.
D20 Maximum # of days with X in any month.
D21 Number of months in which days with X exceeds Y, where Y is the entire visit sample’s average monthly days with X. Calculate Y by averaging over all months with at least one X.
D22 Number of months in which days with X exceeds Y, where Y is person’s average monthly days with X as of this visit. Only consider X that occurred while person was enrolled. If X not observed while enrolled, set to −5.
D23 Number of months in which days with X exceeds Y, where Y is person’s average monthly days with X as of this visit. Only consider X that occurred while person was enrolled prior to visit. If X was not observed during that time, set to 65.
D24 Proportion of months enrolled in which days with X exceeds Y, where Y is entire visit sample’s average monthly days with X. Calculate Y by averaging over all months with at least one X.
D25 Proportion of months enrolled in which days with X exceeds Y, where Y is person’s average monthly days with X as of this visit. Only consider X that occurred while person was enrolled prior to visit up to the full past 60 months.
D26 Total days with X in last month minus monthly average for prior 2–12 months. Only consider X that occurred while person was enrolled prior to visit. If not enrolled ≥2 months, set to −5.
D27 Monthly average of days with X in last 2 months minus monthly average over prior 3–12 months. Only consider X that occurred while person was enrolled prior to visit. If not enrolled ≥3 months, set to −5.
D28 Monthly average of days with X in last 3 months minus monthly average over prior 4–12 months. Only consider X that occurred while person was enrolled prior to visit. If not enrolled ≥4 months, set to −5.

Variables describing monthly occurrence of X in specific time periods:

D29 Number of months with X in last 3 months.
D30 Number of months with X in last 6 months.
D31 Number of months with X in last 12 months.
D32 Number of months with X in last 24 months.
D33 Number of months with X in last 60 months.

Variables describing monthly maxes:

D34 Most recent month in which the maximum (over all 60 months) number of days with X in a month occurred. Use most recent month in case of ties. If X was never observed, assign −5.
D35 Most recent month in which the maximum (over all 60 months) number of days with X in a month occurred. Use most recent month in case of ties. If X was never observed, assign 65.

Variables describing minimum monthly count of X specific time periods:

D36 Minimum monthly count of days with X in last 3 months.
D37 Minimum monthly count of days with X in last 12 months.
D38 Minimum monthly count of days with X in last 24 months.
D39 Minimum monthly count of days with X in last 60 months.
D40 Most recent month in which the minimum (over all 60 months) number of days with X in a month occurred. Use most recent month in case of ties. If X was never observed, assign 0.

Primary reason for MH-related visits. Only calculated for the 18 aforementioned MH diagnosis categories (i.e., not encounters or self-inflicted injury).

D41 Proportion of MH-related visits associated with X during last 1 month, calculated as days with X in last 1 month divided by maximum number of days with any particular diagnosis in last 1 month. If no MH diagnoses in last 1 month, set to −1.
D42 Proportion of MH-related visits associated with X during last 3 months, calculated as days with X in last 3 months divided by maximum number of days with any particular diagnosis in last 3 months. If no MH diagnoses in last 3 months, set to −1.
D43 Proportion of MH-related visits associated with X during last 12 months, calculated as days with X in last 12 months divided by maximum number of days with any particular diagnosis in last 12 months. If no MH diagnoses in last 12 months, set to −1.
D44 Proportion of MH-related visits associated with X during last 24 months, calculated as days with X in last 24 months divided by maximum number of days with any particular diagnosis in last 24 months. If no MH diagnoses in last 24 months, set to −1.
D45 Proportion of MH-related visits associated with X during last 60 months, calculated as days with X in last 60 months divided by maximum number of days with any particular diagnosis in last 60 months. If no MH diagnoses in last 60 months, set to −1.

Variables summarizing 60 months of information on prescription medication fills.

In this section, we use “X” throughout to be consistent and less repetitive, but it should always read “one or more dispensings of prescription drug type X.”

We excluded medications dispensed on the day of the visits in our data pull as information around timing is not sufficient to evaluate if the medication was dispensed before the visit or prescribed during the visit and picked up after the visit finished. We recognize that the days’ supply variable is not ideal but hopefully still informative in this data set.

For each of the following 8 prescription drug types, each of the following variables were computed: antidepressant, benzodiazepine, hypnotic, second generation antipsychotic, first generation antipsychotic, stimulants, lithium, and anticonvulsants. For the full list of medications used in each category see: https://github.com/MHResearchNetwork/more-srpm

Variables summarizing total number (#) of months with X in specific time periods:

1. Binary variable indicating whether X occurred in last 1 month.
2. # of months with X in last 3 months.
3. # of months with X in last 12 months.
4. # of months with X in last 24 months.
5. # of months with X in last 60 months.

Variables describing rate of X in specific time periods while enrolled:

R06 # of months with X in last 3 months divided by # of months enrolled in last 3 months.
R07 # of months with X in last 12 months divided by # of months enrolled in last 12 months.
R08 # of months with X in last 24 months divided by # of months enrolled in last 24 months.
R09 # of months with X in last 60 months divided by # of months enrolled in last 60 months.

Variables describing total days’ supply of X dispensed in specific time periods. Missing days supply will be treated as 0 (i.e., ignored) in all sums.

R10 Total days’ supply of X dispensed in last 1 month.
R11 Total days’ supply of X dispensed in last 3 months.
R12 Total days’ supply of X dispensed in last 12 months.
R13 Total days’ supply of X dispensed in last 24 months.
R14 Total days’ supply of X dispensed in last 60 months.

Variables describing “rate” of days’ supply of X in specific time periods while enrolled. Missing days supply will be treated as 0 (i.e., ignored) in all sums.

R15 Days’ supply of X dispensed in last 3 months divided by # of months enrolled in last 3 months.
R16 Days’ supply of X dispensed in last 12 months divided by # of months enrolled in last 12 months.
R17 Days’ supply of X dispensed in last 24 months divided by # of months enrolled in last 24 months.
R18 Days’ supply of X dispensed sin last 60 months divided by # of months enrolled in last 60 months.

Variables describing timing of X:

R19 Most recent month with X; for those who do not have X (ever), set value for most recent month with X to −5.
R20 Most recent month with X; for those who do not have X (ever), set value for most recent month with X to 65.
R21 First observed month with X; for those who do not have X (ever), set value for most recent month with X to −5.
R22 First observed month with X; for those who do not have X (ever), set value for most recent month with X to 65.

Information on days’ supply of most recent month with X:

R23 Binary indicator for if the person is likely to have drugs on hand the day of the index visit, calculated as days’ supply of most recent month with X divided by 30.4375 minus the # of months ago X occurred. (Yes, this will be a bit crude, but hopefully it will have some predictive power).
R24 Days’ supply of most recent month with X. Those who do not have X (ever), set days’ supply to 0 for those with missing or invalid (i.e., negative) days’ supply values, also set them to zero.

Variables summarizing PHQ responses collected at prior visits or on the day of the index visit.

PHQ information on day of visit:

1. PHQ-8 total score on day of visit. If 5+ items are present, set total score to average of those items multiplied by 8. If <5 items are present, set to −5.
2. PHQ-8 total score on day of visit. If 5+ items are present, set total score to average of those items multiplied by 8. If <5 items are present, set to 35.
3. PHQ item #9 score at visit. If missing, set to −5.
4. PHQ item #9 score at visit. If missing, set to 10.

Prior PHQ item #9 information.

P05 Highest prior PHQ item #9 score. If no prior PHQ item #9 s, set to −5.
P06 Highest prior PHQ item #9 score. If no prior PHQ item #9 s, set to 10.
P07 Number of months (continuous-valued, days / 30.4375) ago an individual had this maximum PHQ item #9 recorded. If never, set to −5.
P08 Number of months (continuous-valued, days / 30.4375) ago an individual had this maximum PHQ item #9 recorded. If never, set to 65.
P09 Number of prior PHQ item #9 s recorded. If none, set to 0.
P10 Number of months (continuous-valued, days / 30.4375) ago an individual last had PHQ item #9 recorded (regardless of its value). If never, set to −5.
P11 Number of months (continuous-valued, days / 30.4375) ago an individual last had PHQ item #9 recorded (regardless of its value). If never, set to 65.

Information about prior recorded PHQ item #9 scores of specific values. Let Y be the PHQ item #9 score that can take on the values 0, 1, 2, and 3.

P12 Number of prior recorded PHQ item #9 scores of Y. If none, set to 0.
P13 Number of prior recorded PHQ item #9 scores of Y while enrolled, divided by number of months enrolled. If none, set to 0.
P14 Number of recorded PHQ item #9 scores of Y in last 1 month. If none, set to 0.
P15 Number of recorded PHQ item #9 scores of Y in last 3 months. If none, set to 0.
P16 Number of recorded PHQ item #9 scores of Y in last 12 months. If none, set to 0.
P17 Number of recorded PHQ item #9 scores of Y in last 24 months. If none, set to 0.
P18 Number of recorded PHQ item #9 scores of Y in last 60 months. If none, set to 0.
P19 Number of recorded PHQ item #9 scores of Y divided by number of recorded PHQ item #9 scores. If none, set to 0.
P20 Number of months (continuous-valued) ago most recent PHQ item #9 score of Y recorded. If never, set to −5.
P21 Number of months (continuous-valued) ago most recent PHQ item #9 score of Y recorded. If never, set to 65.

Information about prior recorded PHQ-8 total scores:

P22 Highest prior observed PHQ-8 total score in past 1 year. If no prior PHQ-8 recorded, set to −5.
P23 Highest prior observed PHQ-8 total score in past 1 year. If no prior PHQ-8 recorded, set to 35.
P24 Highest prior observed PHQ-8 total score in past 2 years. If no prior PHQ-8 recorded, set to −5.
P25 Highest prior observed PHQ-8 total score in past 2 years. If no prior PHQ-8 recorded, set to 35.
P26 Highest prior observed PHQ-8 total score in past 5 years. If no prior PHQ-8 recorded, set to −5.
P27 Highest prior observed PHQ-8 total score in past 5 years. If no prior PHQ-8 recorded, set to 35.
P28 Number of prior recorded PHQ-8 scores above 10. If no prior recorded PHQ-8, set to 0.
P29 Number of months (continuous-valued, days / 30.4375) ago an individual had PHQ-8 score above 10. If never, set to −5.
P30 Number of months (continuous-valued, days / 30.4375) ago an individual had PHQ-8 score above 10. If never, set to 65.
P31 Number prior recorded PHQ-8 scores above 20. If no prior recorded PHQ-8, set to 0.
P32 Number of months (continuous-valued) ago an individual had PHQ-8 score above 20. If never, set to −5.
P33 Number of months (continuous-valued) ago an individual had PHQ-8 score above 20. If never, set to 65.

### Analytic variable (i.e., predictor) specifications for logistic regression (with LASSO)

Predictors based on demographic information:

1. Visit type (MH, general medicine).
2. Age (in years).
3. Sex (Male, Female, Unknown).
4. Race (Asian, Black or African American, Native Hawaiian/Pacific Islander, American Indian/Alaska Native, Multiple races, Other race, Unknown, white).
5. Indicator variable for Hispanic ethnicity.
6. Number of months of prior enrollment.
7. Months since first MH-related visit.
8. Indicator for if census information was available at time of visit.
9. Categorical variable for census information not available, if median household income < $25 K, if median household income ≥ $25 K but < $40 K, or ≥ $40 K.
10. Categorical variable for if census information not available, if neighborhood <25% college-educated, neighborhood ≥25% college-educated
11. Type of insurance coverage (individual binary indicators (not necessarily mutually exclusive as individuals can have multiple coverage) for: Affordable Care Act, Medicaid, commercial, private pay (e.g., individual/family coverage), state-subsidized, self-funded, Medicare, high-deductible, other).
12. Total Charlson score and each of the Charlson subitems. Missing values set to −1, indicating a person did not have any encounters in which to observe diagnoses during 1–365 days prior to visit.

Variables summarizing 60 months of information on diagnoses and utilization.

We use X to denote each diagnosis or utilization type in the descriptions below. We use “X” throughout to be consistent and less repetitive, but it should always read “diagnosis or utilization type X.”

For each of the following 25 categories, the following variables were computed from EHR and insurance claims data:

- *Diagnoses* (18 in total): Depression, anxiety, bipolar, schizophrenia, other psychosis, dementia, attention deficit and hyperactivity disorder (ADHD), Autism spectrum disorder (ASD), personality disorder, alcohol use disorder, drug use disorder, post-traumatic stress disorder (PTSD), eating disorder, traumatic brain injury, conduct/disruptive disorder, diabetes, asthma, pain diagnosis.
- *Mental health-related utilization* (3 types in total): Inpatient encounters with a MH diagnosis, outpatient MH specialty encounters, emergency department encounters with MH diagnosis.
- *Prior injury*, (4 types in total): Any suicide attempt, laceration suicide attempt, other violent suicide attempt, any injury/poisoning diagnosis.

For the full list of ICD-9-CM and ICD-10-CM codes see: https://github.com/MHResearchNetwork/more-srpm

Variables summarizing presence/absence of any relevant diagnoses (from the 18 categories listed above) in specific time periods:

1. Indicator of absence of any MH diagnosis in the last month (i.e., 1 if no MH-related diagnosis in the last month, otherwise 0).
2. Indicator of absence of any MH diagnosis in the last 3 months (i.e., 1 if no MH-related diagnosis in the last month, otherwise 0).
3. Indicator of absence of any MH diagnosis in the last 12 months (i.e., 1 if no MH-related diagnosis in the last month, otherwise 0).
4. Indicator of absence of any MH diagnosis in the last 24 months (i.e., 1 if no MH-related diagnosis in the last month, otherwise 0).
5. Indicator of absence of any MH diagnosis in the last 60 months (i.e., 1 if no MH-related diagnosis in the last month, otherwise 0).

Variables summarizing total count of days with X in specific time periods:

D06 Total count of days with X in last 1 month.
D07 Total count of days with X in last 3 months.
D08 Total count of days with X in last 12 months.
D09 Total count of days with X in last 24 months.
D10 Total count of days with X in last 60 months.
D11 Indicator of absence of diagnosis X at any time in last 60 months i.e., 1 if no diagnosis code found in the last 60 months, otherwise 0.

Variables that describe the past “rate” of X:

D12 Total days with X in last 3 months divided by number of months enrolled in those months.
D13 Total days with X in last 12 months divided by number of months enrolled in those months.
D14 Total days with X in last 24 months divided by number of months enrolled in those months.
D15 Total days with X in the past 60 months divided by number of months enrolled in those months.

Variables capturing information on how recently X occurred:

D16 Most recent occurrence of X (months prior to visit) only for those people with a diagnosis of X at some point, otherwise 0.
D17 Most recent month for which X was not observed only for those people with a diagnosis of X at some point, otherwise 0.

Variables describing earliest occurrence of X:

D18 Earliest occurrence of X (months prior to visit) only for those people with a diagnosis of X at some point, otherwise 0.
D19 Difference between the earliest month and most recent month with occurrence of X only for those people with a diagnosis of X at some point, otherwise 0.

Variables describing trend in X over time:

D20 (# of months with X) × [(difference between the earliest month and most recent month with X] + 1) only for those people with a diagnosis of X at some point, otherwise 0.
D21 Maximum # of days with X in any month minus the minimum count of days with X in any month only for those people with a diagnosis of X at some point, otherwise 0.
D22 Maximum # of days with X in any month only for those people with a diagnosis of X at some point, otherwise 0.
D23 Number of months in which days with X exceeds Y, where Y is the entire visit sample’s average monthly days with X only for those people with a diagnosis of X at some point, otherwise 0. Calculate Y by averaging over all months with at least one X.
D24 Number of months in which days with X exceeds Y, where Y is person’s average monthly days with X as of this visit only for those people with a diagnosis of X at some point, otherwise 0.
D25 Proportion of months enrolled in which days with X exceeds Y, where Y is entire visit sample’s average monthly days with X only for those people with a diagnosis of X at some point, otherwise 0. Calculate Y by averaging over all months with at least one X.
D26 Proportion of months enrolled in which days with X exceeds Y, where Y is person’s average monthly days with X as of this visit. Only consider X that occurred while person was enrolled prior to visit up to the full past 60 months.
D27 Total days with X in last month minus monthly average for prior 2–12 months. Only consider X that occurred while person was enrolled prior to visit. If not enrolled ≥2 months, set to 0.
D28 Monthly average of days with X in last 2 months minus monthly average over prior 3–12 months. Only consider X that occurred while person was enrolled prior to visit. If not enrolled ≥3 months, set to 0.
D29 Monthly average of days with X in last 3 months minus monthly average over prior 4–12 months. Only consider X that occurred while person was enrolled prior to visit. If not enrolled ≥4 months, set to 0.

Variables describing monthly occurrence of X in specific time periods:

D30 Number of months with X in last 3 months.
D31 Number of months with X in last 6 months.
D32 Number of months with X in last 12 months.
D33 Number of months with X in last 24 months.
D34 Number of months with X in last 60 months.

Variables describing monthly maxes:

D35 Most recent month in which the maximum (over all 60 months) number of days with X in a month occurred only for those people with a diagnosis of X at some point, otherwise 0. Use most recent month in case of ties.
  Variables describing minimum monthly count of X specific time periods.
D36 Minimum monthly count of days with X in last 3 months.
D37 Minimum monthly count of days with X in last 12 months.
D38 Minimum monthly count of days with X in last 24 months.
D39 Minimum monthly count of days with X in last 60 months.
D40 Most recent month in which the minimum (over all 60 months) number of days with X in a month occurred. Use most recent month in case of ties. If X was never observed, assign 0.

Primary reason for MH-related visits.

Only calculated for the 18 aforementioned MH diagnosis categories (i.e., not encounters or self-harm injury).

D41 Proportion of MH-related visits associated with X during last 1 month, calculated as days with X in last 1 month divided by maximum number of days with any particular diagnosis in last 1 month. If no MH diagnoses in last 1 month, set to 0.
D42 Proportion of MH-related visits associated with X during last 3 months, calculated as days with X in last 3 months divided by maximum number of days with any particular diagnosis in last 3 months. If no MH diagnoses in last 3 months, set to 0.
D43 Proportion of MH-related visits associated with X during last 12 months, calculated as days with X in last 12 months divided by maximum number of days with any particular diagnosis in last 12 months. If no MH diagnoses in last 12 months, set to 0.
D44 Proportion of MH-related visits associated with X during last 24 months, calculated as days with X in last 24 months divided by maximum number of days with any particular diagnosis in last 24 months. If no MH diagnoses in last 24 months, set to 0.
D45 Proportion of MH-related visits associated with X during last 60 months, calculated as days with X in last 60 months divided by maximum number of days with any particular diagnosis in last 60 months. If no MH diagnoses in last 60 months, set to 0.

Variables summarizing 60 months of information on prescription fills.

In this section, we use “X” throughout to be consistent and less repetitive, but it should always read “one or more dispensings of prescription drug type X.”

We excluded medications dispensed on the day of the visits in our data pull as information around timing is not sufficient to evaluate if the medication was dispensed before the visit or prescribed during the visit and picked up after the visit finished. We recognize that the days’ supply variable is not ideal but hopefully still informative in this data set.

For each of the following 8 prescription drug types, each of the following variables were computed: antidepressant, benzodiazepine, hypnotic, second generation antipsychotic, first generation antipsychotic, stimulants, lithium, and anticonvulsants.

For the full list of medications used in each category see: https://github.com/MHResearchNetwork/more-srpm

Variables summarizing total number (#) of months with X in specific time periods.

1. Binary variable indicating whether X occurred in last 1 month.
2. # of months with X in last 3 months.
3. # of months with X in last 12 months.
4. # of months with X in last 24 months.
5. # of months with X in last 60 months.
6. Indicator for absence of X anytime in the last 60 months (i.e., equal to one if no Rx fill for drug type X, otherwise 0).

Variables describing rate of X in specific time periods while enrolled:

R07 # of months with X in last 3 months divided by # of months enrolled in last 3 months.
R08 # of months with X in last 12 months divided by # of months enrolled in last 12 months.
R09 # of months with X in last 24 months divided by # of months enrolled in last 24 months.
R10 # of months with X in last 60 months divided by # of months enrolled in last 60 months.

Variables describing total days’ supply of X dispensed in specific time periods.

Missing days supply will be treated as 0 (i.e., ignored) in all sums.

R11 Total days’ supply of X dispensed in last 1 month.
R12 Total days’ supply of X dispensed in last 3 months.
R13 Total days’ supply of X dispensed in last 12 months.
R14 Total days’ supply of X dispensed in last 24 months.
R15 Total days’ supply of X dispensed in last 60 months.

Variables describing “rate” of days’ supply of X in specific time periods while enrolled.

Missing days supply will be treated as 0 (i.e., ignored) in all sums.

R16 Days’ supply of X dispensed in last 3 months divided by # of months enrolled in last 3 months.
R17 Days’ supply of X dispensed in last 12 months divided by # of months enrolled in last 12 months.
R18 Days’ supply of X dispensed in last 24 months divided by # of months enrolled in last 24 months.
R19 Days’ supply of X dispensed sin last 60 months divided by # of months enrolled in last 60 months.

Variables describing timing of X:

R20 Most recent month with X for those who have had a script for X ever, otherwise 0.
R21 First observed month with X for those who have had a script for X ever, otherwise 0.

Information on days’ supply of most recent month with X:

R22 Binary indicator for if the person is likely to have drugs on hand the day of the index visit, calculated as days’ supply of most recent month with X divided by 30.4375 minus the # of months ago X occurred.
R23 Days’ supply of most recent month with X. Those who do not have X (ever), set days’ supply to 0.

Variables summarizing PHQ scores collected at prior visits.

PHQ information on day of visit:

1. Indicator for if PHQ-8 missing on the day.
2. Indicator for if PHQ item 9 missing on the day.
3. PHQ-8 total score on day of visit. If 5+ items are present, set total score to average of those items multiplied by 8. If <5 items are present, set to 0.
4. PHQ item 9 score at visit. If missing, set to 0.

Prior PHQ item 9 information:

P05 Indicator for if no prior PHQ item 9 score recorded (i.e., 1 if no prior PHQ item 9 score recorded, if there is one recorded then set to 0).
P06 Highest prior PHQ item 9 score. If no prior PHQ item 9 s, set to 0.
P07 Number of months (continuous-valued, days / 30.4375) ago an individual had this maximum PHQ item 9 recorded. If never, set to 0.
P08 Number of prior PHQ item 9 s recorded. If none, set to 0.
P09 Number of months (continuous-valued, days / 30.4375) ago an individual last had PHQ item 9 recorded (regardless of its value). If never, set to 0.

Information about prior recorded PHQ item 9 scores of specific values. Let Y be the PHQ item#9 score that can take on the values 0, 1, 2, and 3.

P10 Number of prior recorded PHQ item 9 scores of Y. If none, set to 0.
P11 Number of prior recorded PHQ item 9 scores of Y while enrolled, divided by number of months enrolled. If none, set to 0.
P12 Number of recorded PHQ item 9 scores of Y in last 1 month. If none, set to 0.
P13 Number of recorded PHQ item 9 scores of Y in last 3 months. If none, set to 0.
P14 Number of recorded PHQ item 9 scores of Y in last 12 months. If none, set to 0.
P15 Number of recorded PHQ item 9 scores of Y in last 24 months. If none, set to 0.
P16 Number of recorded PHQ item 9 scores of Y in last 60 months. If none, set to 0.
P17 Number of recorded PHQ item 9 scores of Y divided by number of recorded PHQ item 9 scores. If none, set to 0.
P18 Number of months (continuous-valued) ago most recent PHQ item 9 score of Y recorded. If never, set to 0.

Information about prior recorded PHQ-8 total scores:

P19 Highest prior observed PHQ-8 total score in past 1 year. If no prior PHQ-8 recorded, set to 0.
P20 Highest prior observed PHQ-8 total score in past 2 years. If no prior PHQ-8 recorded, set to 0.
P21 Highest prior observed PHQ-8 total score in past 5 years. If no prior PHQ-8 recorded, set to 0.
P22 Number of prior recorded PHQ-8 scores above 10. If no prior recorded PHQ-8, set to 0.
P23 Indicator for if there was no prior PHQ-8 score above 10 (i.e., 1 if had a PHQ-8 above 10, otherwise, including if no prior PHQ-8, 0).
P24 Number of months (continuous-valued, days / 30.4375) ago an individual had PHQ-8 score above 10. If never, set to 0.
P25 Number prior recorded PHQ-8 scores above 20. If no prior recorded PHQ-8, set to 0.
P26 Indicator for if there was no prior PHQ-8 score above 20 (i.e., 1 if had a PHQ-8 above 20, otherwise, including if no prior PHQ-8, 0).
P27 Number of months (continuous-valued) ago an individual had PHQ-8 score above 20. If never, set to 0.

Prespecified interactions for logistic regression models used in screening.

There are already some interactions baked into the variable descriptions above. Here we describe additional interactions that were considered in the lasso model. We created predictors for interactions between clinical information and five variables: age, race and ethnicity, sex, PHQ item 9 response on the day, and prior suicide attempt. In each of the subsections below we describe the clinical information we consider as possible interactions in the logistic regression prediction model.

Due to computational limitations, we were not able to put all interactions into the final model for consideration. We used a screening strategy to whittle these variables down to the number of predictors consider for estimating the lasso model. Below each of the possible interactions are listed by the screening models used. Each screening model was run with lasso with a small tuning parameter to shrink the number of predictors to around 100 predictors per screening model. All of the predictors that made it through this round of screening were then put into the final predictor list for consideration in the final logistic regression with lasso, i.e., these variables were put through a further variable selection process.

Interactions with age groups (in years): 11–17,18–25, 26–35, 36–45, 46–55, 56–65, 66+

- Age screening model 1 interacted age categories with:
  - Diagnosis information: D01, D02, D03, D04, D05, D06, D08, D11, D12, D14,
- Age screening model 2 interacted age categories with:
  - Prior suicide attempt information: D01, D02, D03, D04, D05, D06, D07, D08, D09, D10, D11, D12, D13
  - PHQ-8 total score on the day: P01 and P03
  - Prior PHQ item 9 information: P05, P06, P07, P08, P09, P10, P12, P13, P14, P15, P16, P17, P18
  - PHQ item 9 information on the day: P02, P04
  - Diagnosis timing information: D11, D30, D31, D32, D33, D34, D35

Interactions with race and ethnicity

1. Race and ethnicity screening model 1 interacted race and ethnicity with:
  - Basic diagnosis information: D01, D02, D03, D04, D05, D06, D08, D11, D12, D14
2. Race and ethnicity screening model 2 interacted race and ethnicity with:
  - Medication information: R01 – R22
3. Race and ethnicity screening model 3 interacted race and ethnicity with:
  - Prior suicide attempt information: D01, D02, D03, D04, D05, D06, D07, D08, D09, D10, D11, D12, D13
  - PHQ-8 total score on the day: P01 and P03
  - Prior PHQ item 9 information: P05, P06, P07, P08, P09, P10, P12, P13, P14, P15, P16, P17, P18
  - PHQ item 9 information on the day: P02, P04
  - Diagnosis timing information: D11, D30, D31, D32, D33, D34, D35

Interactions with sex was done with one screening model and included interactions with:

- Diagnosis information: D01, D02, D03, D04, D05, D06, D07, D08, D11, D12, D14, D30, D31, D32, D33, D34, D35.
- Prior suicide attempt information: D01, D02, D03, D04, D05, D06, D07, D08, D09, D10, D11, D12, D13.
- PHQ-8 total score on the day: P01 and P03.
- Prior PHQ item 9 information: P05, P06, P07, P08, P09, P10, P12, P13, P14, P15, P16, P17, P18.
- PHQ item 9 information on the day: P02, P04.

Interactions with PHQ item 9 response on the day was complete in one screening model and included interactions with:

- Diagnosis: D01, D02, D03, D04, D05, D06, D07, D08, D11, D12, D14, D26, D27, D28, D29, D30, D31.
- Prior suicide attempt information: D01, D02, D03, D04, D05, D06, D07, D08, D09, D10, D11, D12, D13.
- Prior PHQ item 9 information: P05, P06, P07, P08, P09, P10, P12, P13, P14, P15, P16, P17, P18.

Interactions with known prior suicide attempt (any known suicide attempt) were fit in one screening model and included interactions with:

- Prior PHQ item 9 information: P05, P06, P07, P08, P09, P10, P12, P13, P14, P15, P16, P17, P18.

### Reporting summary

Further information on research design is available in the Nature Research Reporting Summary linked to this article.

## Supplementary information

Supplementary Info

REPORTING SUMMARY

## Data Availability

The datasets generated and analyzed during this study are not publicly available because they contain detailed information from the electronic health records in the health systems participating in this study and are governed by Health Insurance Portability and Accountability Act (HIPAA). Data are, however, available from the authors upon reasonable request, with permission of all health systems involved and a fully executed data use agreement.

## Code availability

Code used to define predictors and outcomes as well as the national drug code (NDC) list used are available at: https://github.com/MHResearchNetwork/more-srpm. Analytic code for estimating models using all three analytic approaches (logistic regression with lasso, random forest, artificial neural networks) predicting any suicide attempt in the 90 days following an outpatient mental health specialty visit as well as code to evaluate model performance is available at: https://github.com/MHResearchNetwork/NDM_analytic_files.
