# Supplementary material for: Complex modeling with detailed temporal predictors does not improve health records-based suicide risk prediction
Source: NPJ Digit Med. 2023 Mar 23;6:47. doi: 10.1038/s41746-023-00772-4 (PMC10036475; doi:10.1038/s41746-023-00772-4)
Supplement: Supplementary file 1 — Supplementary Info [file 41746_2023_772_MOESM1_ESM.pdf]

Supplementary Information

Additional results to those included in the main text of the manuscript, *Complex modelling with detailed temporal predictors does not improve health records-based suicide risk prediction*. This supplementary material contains the following tables:

Supplementary Table 1. Cohort description of training and validation sample for mental health specialty visits and general medical visits used to estimate suicide death risk prediction model.

Supplementary Table 2. *In-sample* results for 5-fold cross-validation to select tuning parameters for random forest prediction models for suicide attempt (fatal and non-fatal) in the 90 days following the index visit.

Supplementary Table 3. *Out-of-sample* results for 5-fold cross-validation to select tuning parameters for random forest prediction models for suicide attempt (fatal and non-fatal) in the 90 days following the index visit.

Supplementary Table 4. *In-sample* results for 5-fold cross-validation to select tuning parameters for artificial neural network models for suicide attempt (fatal and non-fatal) in the 90 days following the index visit.

Supplementary Table 5. *Out-of-sample* results for 5-fold cross-validation to select tuning parameters for artificial neural network prediction models for suicide attempt (fatal and non-fatal) in the 90 days following the index visit.

Supplementary Table 6. *In-sample* results for 5-fold cross-validation to select tuning parameter for lasso variable selection for logistic regression prediction models for suicide attempt (fatal and non-fatal) in the 90 days following the index visit.

Supplementary Table 7. *Out-of-sample* results for 5-fold cross-validation to select tuning parameter for lasso variable selection for logistic regression prediction models for suicide attempt (fatal and non-fatal) in the 90 days following the index visit.

Supplementary Table 8. Prediction performance in validation data for **suicide attempt in the 90 days** following an outpatient mental health specialty visit across race and ethnicity.

Supplementary Table 9. Prediction performance in validation data for **suicide attempt in the 90 days** following an outpatient mental health specialty visit across sex.

Supplementary Table 10 Prediction performance in validation data for **suicide attempt in the 90 days** following an outpatient general medical visit with a mental health diagnosis across race and ethnicity.

Supplementary Table 11. Prediction performance in validation data for **suicide attempt in the 90 days** following an outpatient general medical visit with a mental health diagnosis across sex.

Supplementary Tables

Supplementary Table 1. **Cohort description of training and validation sample for mental health specialty visits and general medical visits used to estimate suicide death risk prediction model.** *Individuals who reported identifying with more than one listed race and ethnicity contribute to all selected racial and ethnic subgroups. †At least one diagnosis in the last 60 months. ‡At least one prescription filled in the last 60 months.

	Mental health specialty visits				General medical visits			
	Training		Validation		Training		Validation	
Characteristic	N	%	N	%	N	%	N	%
Visits	9,779,178		4,202,240		6,802,509		2,912,308	
People	1,003,508		430,076		1,729,402		741,174	
Visits with a suicide attempt in 90 day	2,234	0.023	965	0.023	1,103	0.016	407	0.014
Female	6,217,808	63.6	2,665,092	63.4	4,262,902	62.7	1,826,417	62.7
Age group (year)								
11-17	1,076,873	11.0	459,214	10.9	421,025	6.2	179,860	6.2
18-29	1,628,136	16.6	707,589	16.8	866,417	12.7	368,574	12.7
30-44	2,487,861	25.4	1,070,197	25.5	1,346,694	19.8	574,469	19.7
45-64	3,469,620	35.5	1,494,362	35.6	2,379,769	35.0	1,014,907	34.8
65 or older	1,116,688	11.4	470,878	11.2	1,788,604	26.3	774,498	26.6
Race and ethnicity*								
Asian	510,800	5.2	218,648	5.2	345,502	5.1	146,063	5.0
American Indian/Alaska Native	95,137	1.0	42,796	1.0	83,545	1.2	29,699	1.0
Black/African American	860,986	8.8	371,438	8.8	522,692	7.7	228,076	7.8
Native Hawaiian/Pacific Islander	103,648	1.1	44,699	1.1	61,746	0.9	27,402	0.9
White, non-Hispanic	5,616,084	57.4	2,410,285	57.4	4,122,681	60.6	1,766,381	60.7
Hispanic ethnicity	2,326,135	23.8	1,000,405	23.8	1,480,345	21.8	633,117	21.7
Not recorded (i.e., race and ethnicity unknown)	383,317	3.9	165,287	3.9	236,469	3.5	103,962	3.6
Insurance Type								
Commercial group	7,201,285	73.6	3,096,273	73.7	4,180,354	61.5	1,782,557	61.2
High-deductible	797,238	8.2	347,339	8.3	446,598	6.6	191,148	6.6
Individual coverage	1,714,131	17.5	731,722	17.4	1,498,960	22.0	648,710	22.3
Medicaid	609,117	6.2	257,590	6.1	586,405	8.6	250,829	8.6
Medicare	1,558,470	15.9	659,764	15.7	1,983,624	29.2	864,062	29.7
PHQ item 9 recorded at index visit	1,299,662	13.3	555,419	13.2	536,331	7.9	231,970	8.0
Response: 0	982,452	10.0	420,254	10.0	427,425	6.3	184,812	6.3
Response: 1	202,508	2.1	86,956	2.1	70,383	1.0	30,231	1.0
Response: 2	67,343	0.7	28,355	0.7	23,616	0.3	10,296	0.4
Response: 3	47,359	0.5	19,854	0.5	14,907	0.2	6,631	0.2
PHQ first 8 items recorded at index vi	1,195,733	12.2	511,756	12.2	538,520	7.9	232,870	8.0
Response: 0-4	284,764	2.9	122,206	2.9	118,339	1.7	50,950	1.7
Response: 5-10	390,844	4.0	168,068	4.0	161,617	2.4	69,801	2.4
Response: 11-15	252,604	2.6	108,103	2.6	126,846	1.9	55,044	1.9
Response: 16-20	177,233	1.8	75,090	1.8	93,168	1.4	40,193	1.4
Response: 21 or higher	90,288	0.9	38,289	0.9	38,550	0.6	16,882	0.6
Anxiety†	6,850,780	70.1	2,947,219	70.1	3,567,884	52.4	1,528,500	52.5
Bipolar†	1,285,117	13.1	551,636	13.1	370,631	5.4	157,865	5.4
Depression†	7,119,353	72.8	3,066,991	73.0	3,850,891	56.6	1,653,667	56.8
Personality disorder†	1,816,326	18.6	784,076	18.7	760,352	11.2	331,131	11.4
Schizophrenia or other psychosis disorder†	762,490	7.8	331,419	7.9	335,122	4.9	146,553	5.0
Traumatic brain injury†	333,691	3.4	141,919	3.4	245,892	3.6	103,848	3.6
Prior mental health inpatient stay‡	2,299,675	23.5	1,000,891	23.8	1,457,470	21.4	633,870	21.8
Prior mental health emergency department visit‡	3,307,818	33.8	1,421,958	33.8	2,078,899	30.6	890,070	30.6
Prior mental health outpatient visit‡	8,942,442	91.4	3,843,978	91.5	3,129,418	46.0	1,341,378	46.1
Prior antidepressant fill‡	6,646,570	68.0	2,858,173	68.0	4,125,466	60.6	1,770,887	60.8
Prior benzodiazepine fill‡	4,510,514	46.1	1,948,438	46.4	2,813,755	41.4	1,201,423	41.3
Prior first generation antipsychotic fil	649,512	6.6	284,828	6.8	399,541	5.9	173,118	5.9
Prior lithium fill‡	405,227	4.1	177,942	4.2	89,229	1.3	40,747	1.4
Prior second generation antipsychotic	2,071,653	21.2	898,982	21.4	649,250	9.5	282,962	9.7

Supplementary Table 2. *In-sample* results averaged over 5-fold cross-validation to select tuning parameters for random forest prediction models for suicide attempt (fatal and non-fatal) in the 90 days following the index visit. AUC = area under the receiver operating curve; PPV = positive predictive value *Unusually longer timing for this setting was due to using a less optimized AWS instance than was used for all other runs.

Tuning parameter values		Performance metrics				
Minimum splitable node size	Number of trees	AUC	Sensitivity, 99th percentile	PPV, 99th percentile	F-score, 99th percentile	Total run time (hours) for cross-validation
Mental health specialty visits						
1,000	10	0.980	0.608	0.387	0.473	6.015
10,000	10	0.939	0.347	0.221	0.270	5.929
25,000	10	0.916	0.278	0.177	0.216	6.277
50,000	10	0.898	0.238	0.151	0.185	6.181
100,000	10	0.879	0.214	0.135	0.166	5.547
1,000	100	0.988	0.668	0.425	0.520	11.242
10,000	100	0.949	0.366	0.233	0.284	10.942
25,000	100	0.926	0.285	0.181	0.221	10.575
50,000	100	0.906	0.245	0.156	0.190	10.132
100,000	100	0.886	0.221	0.141	0.172	14.751*
1,000	500	NA	NA	NA	NA	NA
10,000	500	0.951	0.368	0.234	0.286	36.835
25,000	500	0.928	0.285	0.181	0.222	35.035
50,000	500	0.908	0.245	0.156	0.191	33.191
100,000	500	NA	NA	NA	NA	NA
Mental health visits to a general medical provider						
1,000	10	NA	NA	NA	NA	NA
10,000	10	0.930	0.366	0.120	0.180	3.722
25,000	10	0.906	0.298	0.097	0.147	3.668
50,000	10	0.887	0.263	0.086	0.129	3.543
100,000	10	0.869	0.237	0.077	0.116	3.433
1,000	100	NA	NA	NA	NA	NA
10,000	100	0.942	0.396	0.130	0.195	8.136
25,000	100	0.917	0.321	0.105	0.158	7.919
50,000	100	0.897	0.273	0.089	0.134	7.602
100,000	100	0.878	0.239	0.078	0.118	7.208

Supplementary Table 3. *Out-of-sample* results for 5-fold cross-validation to select tuning parameters for random forest prediction models for suicide attempt (fatal and non-fatal) in the 90 days following the index visit. The bolded row is the optimal tuning parameters selected. AUC = area under the receiver operating curve; PPV = positive predictive value.

Tuning parameter values		Performance metrics			
Minimum splitable node size	Number of trees	AUC	Sensitivity, 99th percentile	PPV, 99th percentile	F-score, 99th percentile
Mental health specialty visits					
1,000	10	0.806	0.153	0.098	0.119
10,000	10	0.835	0.169	0.108	0.132
25,000	10	0.842	0.177	0.113	0.138
50,000	10	0.842	0.180	0.115	0.140
100,000	10	0.841	0.182	0.115	0.141
1,000	100	0.836	0.177	0.112	0.137
10,000	100	0.847	0.187	0.119	0.145
**25,000**	**100**	**0.849**	**0.188**	**0.120**	**0.146**
50,000	100	0.848	0.190	0.121	0.147
100,000	100	0.845	0.189	0.120	0.147
1,000	500	NA	NA	NA	NA
10,000	500	0.848	0.188	0.120	0.147
25,000	500	0.849	0.189	0.120	0.147
50,000	500	0.848	0.190	0.121	0.148
100,000	500	NA	NA	NA	NA
Mental health visits to a general medical provider					
1,000	10	NA	NA	NA	NA
10,000	10	0.824	0.214	0.070	0.106
25,000	10	0.828	0.213	0.070	0.105
50,000	10	0.829	0.213	0.070	0.105
100000	10	0.828	0.212	0.069	0.105
1,000	100	NA	NA	NA	NA
10,000	100	0.835	0.221	0.072	0.109
**25,000**	**100**	**0.836**	**0.219**	**0.072**	**0.108**
50,000	100	0.836	0.219	0.072	0.108
100,000	100	0.833	0.214	0.070	0.105

Supplementary Table 4. *In-sample* results averaged over 5-fold cross-validation to select tuning parameters for artificial neural network models for suicide attempt (fatal and non-fatal) in the 90 days following the index visit. AUC = area under the receiver operating curve; PPV = positive predictive value. *Shorter run times reflect the use of Microsoft Windows Server 2016 on p2.8xlarge (32 vCPUs, 488 GiB memory) instead of the standard g3.16xlarge (64 vCPUs, 488 GiB memory) used in all other cases.

Tuning parameter values		Performance metrics				
Number of hidden layers	Number of nodes per hidden layer	AUC	Sensitivity, 99th percentile	PPV, 99th percentile	F-score, 99th percentile	Total run time (hours) for cross-validation
Mental health specialty visits						
1	4	0.865	0.2195	0.1396	0.171	15.167
1	8	0.869	0.2318	0.1475	0.180	15.500
2	4	0.865	0.2184	0.1390	0.170	18.250
2	8	0.872	0.2453	0.1560	0.191	14.500
2	16	0.878	0.2772	0.1763	0.216	15.000
Mental health visits to a general medical provider						
1	4	0.849	0.2647	0.0870	0.131	8.205*
1	8	0.855	0.2845	0.0934	0.141	8.991*
2	4	0.850	0.2557	0.0840	0.126	8.171*
2	8	0.856	0.2888	0.0949	0.143	8.886*

Supplementary Table 5. *Out-of-sample* results for 5-fold cross-validation to select tuning parameters for artificial neural network prediction models for suicide attempt (fatal and non-fatal) in the 90 days following the index visit. The bolded row is the optimal tuning parameters selected. AUC = area under the receiver operating curve; PPV = positive predictive value.

Tuning parameter values		Performance metrics			
Number of hidden layers	Number of nodes per hidden	AUC	Sensitivity, 99th percentile	PPV, 99th percentile	F-score, 99th percentile
Mental health specialty visits					
**1**	**4**	**0.851**	**0.187**	**0.119**	**0.146**
1	8	0.844	0.180	0.115	0.140
2	4	0.847	0.188	0.119	0.146
2	8	0.842	0.180	0.115	0.140
2	16	0.836	0.171	0.109	0.133
Mental health visits to a general medical provider					
1	4	0.831	0.224	0.074	0.111
1	8	0.814	0.209	0.069	0.103
**2**	**4**	**0.835**	**0.224**	**0.074**	**0.111**
2	8	0.816	0.217	0.071	0.107

Supplementary Table 6. *In-sample* results averaged over 5-fold cross-validation to select tuning parameter for lasso variable selection for logistic regression prediction models for suicide attempt (fatal and non-fatal) in the 90 days following the index visit. AUC = Area under the receiver operating curve; PPV = positive predictive value. *Microsoft Windows Server 2016 on p2.8xlarge (32 vCPUs, 488 GiB memory) used to conduct lasso for the logistic regression, instead of on g3.16xlarge (64 vCPUs, 488 GiB memory).

Tuning parameter value	AUC	Sensitivity, 99th percentile	PPV, 99th percentile	F-score, 99th percentile	Total run time (hours)
Mental health specialty visits					
λ_1 =3.68e-07*	0.864	0.228	0.146	0.178	10.483
λ_2=5.83e-07*	0.864	0.227	0.145	0.177	10.233
λ_3 =9.24e-07*	0.864	0.225	0.144	0.176	10.267
λ_4 =3.68e-06	0.862	0.211	0.135	0.165	15.167
λ_5 =5.83e-06	0.860	0.206	0.131	0.160	16.767
λ_6 =9.24e-06	0.859	0.202	0.129	0.157	15.967
λ_7 =1.46e-05	0.857	0.197	0.126	0.153	15.583
λ_8 =2.32e-05	0.856	0.193	0.124	0.151	15.717
λ_9 =3.68e-05	0.854	0.189	0.121	0.148	14.567
λ_{10} =5.83e-05	0.852	0.185	0.118	0.144	17.083
λ_{11} =9.24e-05	0.849	0.181	0.115	0.141	18.467
λ_{12} =1.46e-04	0.846	0.173	0.111	0.135	17.733
λ_{13} =2.32e-04	0.841	0.166	0.106	0.129	17.200
λ_{14} =3.68e-04	0.830	0.156	0.099	0.121	17.300
λ_{25} =9.24e-04	0.774	0.151	0.071	0.096	16.083
Mental health visits to a general medical provider					
λ_1 =3.68e-07	0.852	0.271	0.089	0.133	12.800
λ_2 =5.83e-07	0.852	0.270	0.088	0.133	13.133
λ_3 =9.24e-07	0.852	0.267	0.087	0.132	11.917
λ_4 =3.68e-06	0.848	0.255	0.083	0.126	11.350
λ_5 =5.83e-06	0.846	0.250	0.082	0.124	11.867
λ_6 =9.24e-06	0.843	0.242	0.079	0.120	11.617
λ_7 =1.46e-05	0.841	0.235	0.077	0.116	11.783
λ_8 =2.32e-05	0.838	0.231	0.076	0.114	11.767
λ_9 =3.68e-05	0.835	0.226	0.074	0.111	11.950
λ_{10} =5.83e-05	0.832	0.217	0.071	0.107	12.117
λ_{11} =9.24e-05	0.828	0.209	0.069	0.103	11.767
λ_{12} =1.46e-04	0.822	0.200	0.066	0.099	11.800
λ_{13} =2.32e-04	0.808	0.190	0.062	0.094	11.567
λ_{14} =3.68e-04	0.784	0.180	0.059	0.089	11.733
λ_{15} =9.24e-04	0.725	0.303	0.019	0.035	11.883

Supplementary Table 7. *Out-of-sample* results for 5-fold cross-validation to select tuning parameter for lasso variable selection for logistic regression prediction models for suicide attempt (fatal and non-fatal) in the 90 days following the index visit. The bolded row is performance of the cross-validation out of sample performance for the tuning parameters selected as optimal. AUC = area under the receiver operating curve; PPV = positive predictive value

Tuning parameter value	AUC	Sensitivity, 99th percentile	PPV, 99th percentile	F-score, 99th percentile
Mental health specialty visits				
λ_1 =3.68e-07	0.850	0.187	0.120	0.146
λ_2 =5.83e-07	0.850	0.187	0.120	0.146
λ_3 =9.24e-07	0.851	0.188	0.120	0.146
λ_4 =3.68e-06	0.852	0.187	0.120	0.146
λ_5 =5.83e-06	0.852	0.188	0.120	0.147
λ_6 =9.24e-06	0.853	0.187	0.120	0.146
**λ_7 =1.46e-05**	**0.853**	**0.187**	**0.119**	**0.146**
λ_8 =2.32e-05	0.852	0.186	0.119	0.145
λ_9 =3.68e-05	0.851	0.182	0.116	0.142
λ_{10} =5.83e-05	0.850	0.18	0.115	0.140
λ_{11} =9.24e-05	0.847	0.177	0.113	0.138
λ_{12} =1.46e-04	0.845	0.17	0.109	0.133
λ_{13} =2.32e-04	0.840	0.164	0.105	0.128
λ_{14} =3.68e-04	0.829	0.153	0.098	0.120
λ_{25} =9.24e-04	0.772	0.118	0.070	0.088
Mental health visits to a general medical provider				
λ_1 =3.68e-07	0.832	0.223	0.073	0.110
λ_2 =5.83e-07	0.833	0.225	0.074	0.111
λ_3 =9.24e-07	0.835	0.227	0.074	0.112
λ_4 =3.68e-06	0.836	0.229	0.075	0.113
λ_5 =5.83e-06	0.836	0.228	0.075	0.112
**λ_6 =9.24e-06**	**0.837**	**0.226**	**0.074**	**0.111**
λ_7 =1.46e-05	0.836	0.224	0.073	0.111
λ_8 =2.32e-05	0.835	0.224	0.073	0.110
λ_9 =3.68e-05	0.832	0.221	0.072	0.109
λ_{10} =5.83e-05	0.830	0.213	0.070	0.105
λ_{11} =9.24e-05	0.826	0.206	0.067	0.101
λ_{12} =1.46e-04	0.821	0.195	0.064	0.096
λ_{13} =2.32e-04	0.807	0.188	0.062	0.093
λ_{14} =3.68e-04	0.783	0.180	0.059	0.089
λ_{15} =9.24e-04	0.720	0.048	0.015	0.023

Supplementary Table 8. Prediction performance in validation data for **suicide attempt in the 90 days** following an outpatient mental health specialty visit across race and ethnicity; 95% confidence intervals constructed using 10,000 bootstrap samples. AUC = area under the receiver operating curve; PPV = positive predictive value; LR=logistic regression (after lasso variable selection); RF=random forests; ANN=artificial neural network.

Prediction Model	AUC (95% CI)	Brier score (95% CI)	F-score, 99th percentile (95% CI)	Sensitivity, 99th percentile (95% CI)	Specificity, 99th percentile (95% CI)	PPV, 99th percentile (95% CI)
America Indian / Alaskan Native (N visits (events) training: 105,359 (851); N visits (events) testing: 43,814 (350))						
OP	0.835	$7.8x10^{-3}$	0.143	0.203	0.987	0.111
	(0.813, 0.855)	(7.0,8.6) $x10^{-3}$	(0.114, 0.173)	(0.161, 0.245)	(0.986, 0.988)	(0.087, 0.136)
LR	0.834	$7.8x10^{-3}$	0.161	0.211	0.989	0.131
	(0.811, 0.856)	(7.0,8.5) $x10^{-3}$	(0.129, 0.193)	(0.169, 0.254)	(0.988, 0.990)	(0.103, 0.159)
RF	0.823	$7.6x10^{-3}$	0.130	0.183	0.987	0.100
	(0.800, 0.846)	(6.9,8.4) $x10^{-3}$	(0.101, 0.159)	(0.142, 0.224)	(0.986, 0.988)	(0.078, 0.125)
ANN	0.844	$7.5x10^{-3}$	0.156	0.194	0.990	0.130
	(0.823, 0.864)	(6.8,8.3) $x10^{-3}$	(0.123, 0.189)	(0.153, 0.237)	(0.989, 0.991)	(0.102, 0.160)
Ensemble:	0.834	$7.5x10^{-3}$	0.171	0.226	0.989	0.138
LR/RF	(0.811, 0.856)	(6.8,8.3) $x10^{-3}$	(0.138, 0.204)	(0.182, 0.270)	(0.988, 0.990)	(0.110, 0.167)
Ensemble:	0.836	$7.6x10^{-3}$	0.150	0.206	0.988	0.118
RF/ANN	(0.814, 0.858)	(6.8,8.3) $x10^{-3}$	(0.119, 0.181)	(0.163, 0.249)	(0.987, 0.989)	(0.093, 0.145)
Ensemble:	0.842	$7.6x10^{-3}$	0.172	0.223	0.989	0.140
LR/ANN	(0.820, 0.863)	(6.8,8.4) $x10^{-3}$	(0.138, 0.205)	(0.179, 0.267)	(0.988, 0.990)	(0.111, 0.169)
Ensemble:	0.839	$7.5x10^{-3}$	0.169	0.223	0.989	0.136
LR/RF/ANN	(0.816, 0.860)	(6.8,8.3) $x10^{-3}$	(0.136, 0.202)	(0.179, 0.267)	(0.988, 0.990)	(0.109, 0.165)
Asian (N visits (events) training: 575,790 (3,343); N visits (events) testing: 241,079 (1,356))						
OP	0.861	$5.5x10^{-3}$	0.131	0.159	0.993	0.111
	(0.850, 0.871)	(5.2,5.7) $x10^{-3}$	(0.115, 0.146)	(0.140, 0.179)	(0.992, 0.993)	(0.097, 0.125)
LR	0.859	$5.5x10^{-3}$	0.133	0.173	0.992	0.108
	(0.848, 0.869)	(5.2,5.8) $x10^{-3}$	(0.118, 0.149)	(0.153, 0.193)	(0.992, 0.992)	(0.096, 0.122)
RF	0.845	$5.5x10^{-3}$	0.103	0.131	0.992	0.085
	(0.834, 0.856)	(5.2,5.8) $x10^{-3}$	(0.089, 0.117)	(0.113, 0.149)	(0.992, 0.992)	(0.073, 0.097)
ANN	0.858	$5.5x10^{-3}$	0.126	0.149	0.993	0.108
	(0.848, 0.868)	(5.2,5.7) $x10^{-3}$	(0.110, 0.141)	(0.130, 0.168)	(0.993, 0.993)	(0.094, 0.123)
Ensemble:	0.859	$5.5x10^{-3}$	0.125	0.162	0.992	0.103
LR/RF	(0.848, 0.869)	(5.2,5.7) $x10^{-3}$	(0.111, 0.140)	(0.142, 0.181)	(0.992, 0.992)	(0.090, 0.115)
Ensemble:	0.857	$5.5x10^{-3}$	0.121	0.151	0.992	0.102
RF/ANN	(0.847, 0.867)	(5.2,5.7) $x10^{-3}$	(0.106, 0.137)	(0.132, 0.171)	(0.992, 0.993)	(0.088, 0.115)
Ensemble:	0.860	$5.5x10^{-3}$	0.136	0.173	0.992	0.112
LR/ANN	(0.850, 0.870)	(5.2,5.7) $x10^{-3}$	(0.121, 0.152)	(0.153, 0.194)	(0.992, 0.993)	(0.099, 0.126)
Ensemble:	0.861	$5.5x10^{-3}$	0.127	0.162	0.992	0.105
LR/RF/ANN	(0.850, 0.871)	(5.2,5.7) $x10^{-3}$	(0.112, 0.142)	(0.143, 0.182)	(0.992, 0.993)	(0.092, 0.118)
Black / African American:(N visits (events) training: 937,826 (4,327); N visits (events) testing: 400,089 (2,067))						
OP	0.837	$5.1x10^{-3}$	0.095	0.097	0.995	0.092
	(0.828, 0.845)	(4.9,5.3) $x10^{-3}$	(0.083, 0.107)	(0.084, 0.110)	(0.995, 0.995)	(0.080, 0.105)
LR	0.831	$5.0x10^{-3}$	0.100	0.110	0.994	0.092
	(0.822, 0.839)	(4.8,5.3) $x10^{-3}$	(0.088, 0.112)	(0.097, 0.124)	(0.994, 0.995)	(0.080, 0.103)
RF	0.824	$5.0x10^{-3}$	0.104	0.122	0.994	0.091
	(0.815, 0.833)	(4.8,5.2) $x10^{-3}$	(0.092, 0.116)	(0.108, 0.136)	(0.993, 0.994)	(0.081, 0.102)
ANN	0.837	$5.0x10^{-3}$	0.116	0.121	0.995	0.112
	(0.828, 0.845)	(4.8,5.2) $x10^{-3}$	(0.103, 0.129)	(0.107, 0.135)	(0.995, 0.995)	(0.099, 0.125)
Ensemble:	0.836	$5.0x10^{-3}$	0.110	0.121	0.994	0.100
LR/RF	(0.827, 0.844)	(4.8,5.2) $x10^{-3}$	(0.097, 0.122)	(0.107, 0.136)	(0.994, 0.995)	(0.089, 0.112)
Ensemble:	0.837	$5.0x10^{-3}$	0.106	0.115	0.994	0.098
RF/ANN	(0.829, 0.846)	(4.8,5.2) $x10^{-3}$	(0.094, 0.118)	(0.102, 0.129)	(0.994, 0.995)	(0.086, 0.110)
Ensemble:	0.836	$5.0x10^{-3}$	0.105	0.114	0.995	0.097
LR/ANN	(0.828, 0.844)	(4.8,5.2) $x10^{-3}$	(0.092, 0.117)	(0.100, 0.127)	(0.994, 0.995)	(0.086, 0.109)
Ensemble:	0.839	$5.0x10^{-3}$	0.105	0.114	0.995	0.098
LR/RF/ANN	(0.831, 0.847)	(4.8,5.2) $x10^{-3}$	(0.093, 0.118)	(0.101, 0.128)	(0.994, 0.995)	(0.086, 0.110)

Supplementary Table 8 continued. Prediction performance in validation data for **suicide attempt in the 90 days** following an outpatient mental health specialty visit across race and ethnicity; 95% confidence intervals constructed using 10,000 bootstrap samples. AUC = area under the receiver operating curve; PPV = positive predictive value; LR=logistic regression (after lasso variable selection); RF=random forests; ANN=artificial neural network.

Prediction Model	AUC (95% CI)	Brier score (95% CI)	F-score, 99th percentile (95% CI)	Sensitivity, 99th percentile (95% CI)	Specificity, 99th percentile (95% CI)	PPV, 99th percentile (95% CI)
Native Hawaiian / Pacific Islander (N visits (events) training: 112,581 (732); N visits (events) testing: 48,880 (296))						
OP	0.830	$5.8x10^{-3}$	0.145	0.155	0.994	0.135
	(0.808, 0.851)	$(5.2,6.5) x10^{-3}$	(0.108, 0.182)	(0.115, 0.197)	(0.993, 0.995)	(0.100, 0.172)
LR	0.843	$5.8x10^{-3}$	0.144	0.189	0.991	0.117
	(0.820, 0.865)	$(5.2,6.4) x10^{-3}$	(0.112, 0.178)	(0.146, 0.234)	(0.990, 0.992)	(0.089, 0.146)
RF	0.805	$5.9x10^{-3}$	0.089	0.081	0.996	0.100
	(0.778, 0.831)	$(5.3,6.6) x10^{-3}$	(0.057, 0.123)	(0.051, 0.113)	(0.995, 0.996)	(0.064, 0.139)
ANN	0.839	$5.8x10^{-3}$	0.160	0.172	0.994	0.149
	(0.816, 0.862)	$(5.2,6.4) x10^{-3}$	(0.121, 0.198)	(0.130, 0.217)	(0.993, 0.995)	(0.112, 0.188)
Ensemble:	0.833	$5.8x10^{-3}$	0.139	0.166	0.993	0.120
LR/RF	(0.809, 0.856)	$(5.2,6.4) x10^{-3}$	(0.105, 0.174)	(0.125, 0.209)	(0.992, 0.993)	(0.090, 0.152)
Ensemble:	0.830	$5.8x10^{-3}$	0.139	0.142	0.995	0.136
RF/ANN	(0.807, 0.853)	$(5.2,6.5) x10^{-3}$	(0.102, 0.177)	(0.104, 0.183)	(0.994, 0.995)	(0.099, 0.176)
Ensemble:	0.844	$5.8x10^{-3}$	0.151	0.186	0.992	0.127
LR/ANN	(0.821, 0.866)	$(5.1,6.4) x10^{-3}$	(0.116, 0.187)	(0.143, 0.231)	(0.991, 0.993)	(0.097, 0.160)
Ensemble:	0.838	$5.8x10^{-3}$	0.143	0.169	0.993	0.124
LR/RF/ANN	(0.815, 0.860)	$(5.2,6.4) x10^{-3}$	(0.108, 0.178)	(0.128, 0.212)	(0.992, 0.993)	(0.093, 0.157)
Non-Hispanic white (N visits (events) training: 6,130,341 (42,028); N visits (events) testing: 2,630,268 (18,569))						
OP	0.852	$6.8x10^{-3}$	0.124	0.165	0.989	0.100
	(0.849, 0.854)	$(6.7,6.9) x10^{-3}$	(0.120, 0.128)	(0.159, 0.170)	(0.989, 0.990)	(0.097, 0.103)
LR	0.856	$6.8x10^{-3}$	0.132	0.187	0.988	0.102
	(0.853, 0.859)	$(6.7,6.9) x10^{-3}$	(0.128, 0.136)	(0.181, 0.193)	(0.988, 0.988)	(0.099, 0.106)
RF	0.851	$6.8x10^{-3}$	0.139	0.181	0.990	0.112
	(0.849, 0.854)	$(6.7,6.9) x10^{-3}$	(0.134, 0.143)	(0.176, 0.187)	(0.990, 0.990)	(0.108, 0.116)
ANN	0.857	$6.8x10^{-3}$	0.137	0.177	0.990	0.112
	(0.855, 0.860)	$(6.7,6.9) x10^{-3}$	(0.133, 0.142)	(0.171, 0.182)	(0.990, 0.990)	(0.109, 0.116)
Ensemble:	0.862	$6.8x10^{-3}$	0.138	0.190	0.989	0.108
LR/RF	(0.859, .865)	$(6.7,6.9) x10^{-3}$	(0.133, 0.142)	(0.184, 0.195)	(0.989, 0.989)	(0.105, 0.111)
Ensemble:	0.862	$6.8x10^{-3}$	0.140	0.181	0.990	0.114
RF/ANN	(0.859, 0.864)	$(6.7,6.9) x10^{-3}$	(0.136, 0.144)	(0.176, 0.187)	(0.990, 0.990)	(0.111, 0.118)
Ensemble:	0.859	$6.8x10^{-3}$	0.136	0.185	0.989	0.107
LR/ANN	(0.856, 0.862)	$(6.7,6.9) x10^{-3}$	(0.131, 0.140)	(0.180, 0.191)	(0.989, 0.989)	(0.103, 0.110)
Ensemble:	0.863	$6.8x10^{-3}$		0.189	0.989	0.110
LR/RF/ANN	(0.861, 0.866)	$(6.7,6.9) x10^{-3}$	0.139 (0.135, 0.14	(0.183, 0.195)	(0.989, 0.989)	(0.107, 0.114)
Hispanic (N visits (events) training: 2,589,816 (16,293); N visits (events) testing: 1,120,187 (6,991))						
OP	0.848	$6.0x10^{-3}$		0.173	0.992	0.121
	(0.844, 0.852)	$(5.9,6.1) x10^{-3}$	0.142 (0.135, 0.14	(0.164, 0.182)	(0.992, 0.992)	(0.114, 0.127)
LR	0.845	$6.1x10^{-3}$		0.187	0.990	0.108
	(0.840, 0.849)	$(6.0,6.2) x10^{-3}$	0.137 (0.131, 0.14	(0.178, 0.197)	(0.990, 0.990)	(0.103, 0.114)
RF	0.844	$6.0x10^{-3}$		0.175	0.992	0.116
	(0.839, 0.849)	$(5.9,6.1) x10^{-3}$	0.140 (0.133, 0.14	(0.166, 0.184)	(0.991, 0.992)	(0.110, 0.122)
ANN	0.849	$6.0x10^{-3}$		0.180	0.991	0.114
	(0.844, 0.853)	$(5.9,6.1) x10^{-3}$	0.140 (0.133, 0.14	(0.171, 0.189)	(0.991, 0.991)	(0.108, 0.120)
Ensemble:	0.853	$6.0x10^{-3}$		0.188	0.991	0.114
LR/RF	(0.849, 0.858)	$(5.8,6.1) x10^{-3}$	0.142 (0.135, 0.14	(0.178, 0.197)	(0.991, 0.991)	(0.108, 0.120)
Ensemble:	0.853	$6.0x10^{-3}$		0.183	0.991	0.118
RF/ANN	(0.849, 0.857)	$(5.9,6.1) x10^{-3}$	0.144 (0.136, 0.15	(0.174, 0.192)	(0.991, 0.992)	(0.112, 0.124)
Ensemble:	0.850	$6.0x10^{-3}$		0.191	0.991	0.113
LR/ANN	(0.845, 0.854)	$(5.9,6.1) x10^{-3}$	0.142 (0.135, 0.14	(0.182, 0.200)	(0.990, 0.991)	(0.107, 0.119)
Ensemble:	0.854	$6.0x10^{-3}$		0.189	0.991	0.115
LR/RF/ANN	(0.850, 0.859)	$(5.8,6.1) x10^{-3}$	0.143 (0.136, 0.15	(0.180, 0.198)	(0.991, 0.991)	(0.110, 0.121)

Supplementary Table 8 continued. Prediction performance in validation data for **suicide attempt in the 90 days** following an outpatient mental health specialty visit across race and ethnicity; 95% confidence intervals constructed using 10,000 bootstrap samples. AUC = area under the receiver operating curve; PPV = positive predictive value; LR=logistic regression (after lasso variable selection); RF=random forests; ANN=artificial neural network.

Prediction Model	AUC (95% CI)	Brier score (95% CI)	F-score, 99th percentile (95% CI)	Sensitivity, 99th percentile (95% CI)	Specificity, 99th percentile (95% CI)	PPV, 99th percentile (95% CI)
Race Unknown (N visits (events) training: 356,691 (1,628); N visits (events) testing: 147,921 (663))						
OP	0.755<br>(0.734, 0.775)	4.5×10^{-3}<br>$(4.1,4.8) \times 10^{-3}$	0.054<br>(0.037, 0.072)	0.048<br>(0.033, 0.065)	0.997<br>(0.996, 0.997)	0.061<br>(0.042, 0.081)
LR	0.775<br>(0.756, 0.794)	4.5×10^{-3}<br>$(4.1,4.8) \times 10^{-3}$	0.068<br>(0.050, 0.087)	0.068<br>(0.050, 0.087)	0.996<br>(0.996, 0.996)	0.069<br>(0.050, 0.088)
RF	0.788<br>(0.769, 0.805)	4.4×10^{-3}<br>$(4.1,4.7) \times 10^{-3}$	0.074<br>(0.055, 0.094)	0.071<br>(0.052, 0.091)	0.996<br>(0.996, 0.996)	0.077<br>(0.057, 0.099)
ANN	0.770<br>(0.751, 0.789)	4.4×10^{-3}<br>$(4.1,4.7) \times 10^{-3}$	0.086<br>(0.064, 0.108)	0.078<br>(0.059, 0.100)	0.997<br>(0.996, 0.997)	0.095<br>(0.071, 0.121)
Ensemble: LR/RF	0.797<br>(0.779, 0.813)	4.4×10^{-3}<br>$(4.1,4.8) \times 10^{-3}$	0.087<br>(0.066, 0.109)	0.081<br>(0.061, 0.102)	0.996<br>(0.996, 0.997)	0.094<br>(0.071, 0.118)
Ensemble: RF/ANN	0.789<br>(0.771, 0.806)	4.4×10^{-3}<br>$(4.1,4.7) \times 10^{-3}$	0.077<br>(0.057, 0.099)	0.069<br>(0.051, 0.089)	0.997<br>(0.996, 0.997)	0.087<br>(0.064, 0.112)
Ensemble: LR/ANN	0.775<br>(0.756, 0.794)	4.4×10^{-3}<br>$(4.1,4.8) \times 10^{-3}$	0.072<br>(0.053, 0.092)	0.069<br>(0.051, 0.089)	0.996<br>(0.996, 0.996)	0.075<br>(0.055, 0.097)
Ensemble: LR/RF/ANN	0.792<br>(0.774, 0.809)	4.4×10^{-3}<br>$(4.1,4.7) \times 10^{-3}$	0.086<br>(0.065, 0.108)	0.080<br>(0.060, 0.101)	0.996<br>(0.996, 0.997)	0.093<br>(0.070, 0.117)

Supplementary Table 9. Prediction performance in validation data for **suicide attempt in the 90 days** following an outpatient mental health specialty visit across sex; 95% confidence intervals constructed using 10,000 bootstrap samples. AUC = area under the receiver operating curve; PPV = positive predictive value; LR=logistic regression (after lasso variable selection); RF=random forests; ANN=artificial neural network

Prediction Model	AUC (95% CI)	Brier score (95% CI)	F-score, 99th percentile (95% CI)	Sensitivity, 99th percentile (95% CI)	Specificity, 99th percentile (95% CI)	PPV, 99th percentile (95% CI)
Female (N visits (events) training: 6,809,585 (46,636); N visits (events) testing: 2,917,535 (20,790))						
OP	0.858	6.9x10^{-3}	0.137	0.186	0.989	0.108
	(0.855, 0.861)	(6.8,7.0) x10^{-3}	(0.133, 0.141)	(0.181, 0.191)	(0.989, 0.989)	(0.105, 0.112)
LR	0.858	6.9x10^{-3}	0.141	0.202	0.988	0.109
	(0.855, 0.860)	(6.8,7.0) x10^{-3}	(0.138, 0.145)	(0.197, 0.207)	(0.988, 0.988)	(0.106, 0.112)
RF	0.853	6.8x10^{-3}	0.146	0.191	0.990	0.118
	(0.851, 0.856)	(6.7,7.0) x10^{-3}	(0.142, 0.150)	(0.186, 0.196)	(0.990, 0.990)	(0.115, 0.122)
ANN	0.860	6.8x10^{-3}	0.147	0.195	0.989	0.118
	(0.857, 0.862)	(6.7,6.9) x10^{-3}	(0.143, 0.151)	(0.190, 0.201)	(0.989, 0.990)	(0.114, 0.121)
Ensemble:	0.863	6.8x10^{-3}	0.147	0.204	0.989	0.115
LR/RF	(0.861, 0.866)	(6.7,6.9) x10^{-3}	(0.144, 0.151)	(0.199, 0.210)	(0.989, 0.989)	(0.112, 0.119)
Ensemble:	0.863	6.8x10^{-3}	0.149	0.196	0.990	0.120
RF/ANN	(0.861, 0.866)	(6.7,6.9) x10^{-3}	(0.145, 0.153)	(0.190, 0.201)	(0.990, 0.990)	(0.116, 0.123)
Ensemble:	0.861	6.8x10^{-3}	0.145	0.203	0.989	0.113
LR/ANN	(0.858, 0.864)	(6.7,6.9) x10^{-3}	(0.141, 0.149)	(0.198, 0.208)	(0.988, 0.989)	(0.110, 0.116)
Ensemble:	0.865	6.8x10^{-3}		0.204	0.989	0.117
LR/RF/ANN	(0.862, 0.867)	(6.7,6.9) x10^{-3}	0.149 (0.145, 0.1	(0.199, 0.210)	(0.989, 0.989)	(0.114, 0.120)
Male (N visits (events) training: 3,863,780 (21,543); N visits (events) testing: 1,657,088 (9,120))						
OP	0.825	5.4x10^{-3}	0.095	0.100	0.994	0.091
	(0.821, 0.829)	(5.3,5.5) x10^{-3}	(0.089, 0.101)	(0.094, 0.106)	(0.994, 0.995)	(0.085, 0.096)
LR	0.832	5.4x10^{-3}	0.106	0.132	0.993	0.089
	(0.828, 0.837)	(5.3,5.5) x10^{-3}	(0.101, 0.112)	(0.125, 0.139)	(0.992, 0.993)	(0.084, 0.094)
RF	0.831	5.4x10^{-3}	0.107	0.128	0.993	0.092
	(0.827, 0.835)	(5.3,5.5) x10^{-3}	(0.101, 0.112)	(0.121, 0.135)	(0.993, 0.993)	(0.087, 0.097)
ANN	0.836	5.4x10^{-3}	0.107	0.118	0.994	0.098
	(0.831, 0.840)	(5.2,5.5) x10^{-3}	(0.101, 0.113)	(0.111, 0.125)	(0.994, 0.994)	(0.092, 0.103)
Ensemble:	0.841	5.4x10^{-3}	0.110	0.134	0.993	0.093
LR/RF	(0.837, 0.845)	(5.4,5.5) x10^{-3}	(0.104, 0.115)	(0.127, 0.141)	(0.993, 0.993)	(0.088, 0.098)
Ensemble:	0.841	5.3x10^{-3}	0.110	0.126	0.994	0.097
RF/ANN	(0.837, 0.845)	(5.2,5.5) x10^{-3}	(0.104, 0.116)	(0.119, 0.133)	(0.993, 0.994)	(0.092, 0.103)
Ensemble:	0.837	5.4x10^{-3}	0.108	0.128		0.094
LR/ANN	(0.832, 0.841)	(5.3,5.5) x10^{-3}	(0.103, 0.114)	(0.121, 0.135)	0.993 (0.993, 0.9	(0.089, 0.099)
Ensemble:	0.843	5.3x10^{-3}	0.111	0.131		0.095
LR/RF/ANN	(0.839, 0.847)	(5.2,5.5) x10^{-3}	(0.105, 0.116)	(0.125, 0.138)	0.993 (0.993, 0.9	(0.090, 0.101)

Supplementary Table 10 Prediction performance in validation data for **suicide attempt in the 90 days** following an outpatient general medical visit with a mental health diagnosis across race and ethnicity; 95% confidence intervals constructed using 10,000 bootstrap samples. AUC = Area under the receiver operating curve; PPV = Positive predicted value; OP: original parsimonious; LR=Logistic regression with Lasso variable selection; RF = random forest; ANN = artificial neural network

Prediction Model	AUC (95% CI)	Brier score (95% CI)	F-score, 99th percentile (95% CI)	Sensitivity, 99th percentile (95% CI)	Specificity, 99th percentile (95% CI)	PPV, 99th percentile (95% CI)
America Indian / Alaskan Native (N visits (events) training: 83,460 (399); N visits (events) testing: 37,278 (233))						
OP	0.820	$6.1x10^{-3}$	0.099	0.193	0.983	0.067
	(0.789, 0.850)	($5.4x10^{-3}$, $6.9x10^{-3}$)	(0.073, 0.127)	(0.143, 0.245)	(0.982, 0.984)	(0.048, 0.086)
LR	0.831	$6.1x10^{-3}$	0.083	0.176	0.981	0.055
	(0.807, 0.853)	($5.4x10^{-3}$, $6.9x10^{-3}$)	(0.059, 0.107)	(0.128, 0.226)	(0.979, 0.982)	(0.039, 0.071)
RF	0.847	$6.1x10^{-3}$	0.074	0.137	0.984	0.051
	(0.821, 0.872)	($5.3x10^{-3}$, $6.9x10^{-3}$)	(0.050, 0.098)	(0.093, 0.182)	(0.982, 0.985)	(0.034, 0.068)
ANN	0.857	$6.1x10^{-3}$	0.081	0.163	0.982	0.054
	(0.832, 0.880)	($5.3x10^{-3}$, $6.9x10^{-3}$)	(0.057, 0.105)	(0.116, 0.212)	(0.981, 0.983)	(0.037, 0.070)
Ensemble:	0.853	$6.1x10^{-3}$	0.070	0.142	0.982	0.046
LR/RF	(0.829, 0.876)	($5.3x10^{-3}$, $6.9x10^{-3}$)	(0.047, 0.092)	(0.097, 0.187)	(0.980, 0.983)	(0.031, 0.062)
Ensemble:	0.855	$6.1x10^{-3}$	0.088	0.176	0.982	0.058
RF/ANN	(0.830, 0.880)	($5.3x10^{-3}$, $6.9x10^{-3}$)	(0.063, 0.113)	(0.127, 0.226)	(0.981, 0.984)	(0.042, 0.076)
Ensemble:	0.850	$6.1x10^{-3}$	0.084	0.176	0.981	0.055
LR/ANN	(0.826, 0.872)	($5.3x10^{-3}$, $6.9x10^{-3}$)	(0.060, 0.108)	(0.127, 0.226)	(0.980, 0.982)	(0.039, 0.072)
Ensemble:	0.855	$6.1x10^{-3}$	0.081	0.163	0.982	0.054
LR/RF/ANN	(0.830, 0.879)	($5.3x10^{-3}$, $6.9x10^{-3}$)	(0.057, 0.105)	(0.116, 0.211)	(0.981, 0.983)	(0.038, 0.071)
Asian (N visits (events) training: 377,044 (890); N visits (events) testing: 163,118 (417))						
OP	0.824	$2.5x10^{-3}$	0.100	0.189	0.993	0.068
	(0.801, 0.847)	($2.3x10^{-3}$, $2.8x10^{-3}$)	(0.080, 0.121)	(0.152, 0.229)	(0.993, 0.994)	(0.054, 0.083)
LR	0.821	$2.5x10^{-3}$	0.097	0.177	0.994	0.067
	(0.798, 0.843)	($2.3x10^{-3}$, $2.8x10^{-3}$)	(0.077, 0.118)	(0.141, 0.215)	(0.993, 0.994)	(0.053, 0.082)
RF	0.817	$2.5x10^{-3}$	0.083	0.173	0.992	0.055
	(0.795, 0.840)	($2.3x10^{-3}$, $2.8x10^{-3}$)	(0.066, 0.102)	(0.137, 0.209)	(0.992, 0.993)	(0.043, 0.068)
ANN	0.819	$2.5x10^{-3}$	0.103	0.197	0.993	0.070
	(0.796, 0.842)	($2.3x10^{-3}$, $2.7x10^{-3}$)	(0.083, 0.124)	(0.159, 0.236)	(0.993, 0.994)	(0.056, 0.085)
Ensemble:	0.826	$2.5x10^{-3}$	0.093	0.182	0.993	0.062
LR/RF	(0.804, 0.848)	($2.3x10^{-3}$, $2.8x10^{-3}$)	(0.074, 0.113)	(0.146, 0.220)	(0.993, 0.993)	(0.049, 0.076)
Ensemble:	0.825	$2.5x10^{-3}$	0.100	0.201	0.993	0.066
RF/ANN	(0.803, 0.847)	($2.3x10^{-3}$, $2.7x10^{-3}$)	(0.081, 0.120)	(0.164, 0.241)	(0.992, 0.993)	(0.053, 0.080)
Ensemble:	0.822	$2.5x10^{-3}$	0.099	0.187	0.993	0.068
LR/ANN	(0.799, 0.844)	($2.3x10^{-3}$, $2.8x10^{-3}$)	(0.079, 0.120)	(0.150, 0.225)	(0.993, 0.994)	(0.054, 0.083)
Ensemble:	0.827	$2.5x10^{-3}$	0.095	0.185	0.993	0.064
LR/RF/ANN	(0.805, 0.849)	($2.3x10^{-3}$, $2.7x10^{-3}$)	(0.076, 0.115)	(0.148, 0.222)	(0.993, 0.993)	(0.051, 0.078)
Black / African American (N visits (events) training: 586,268 (1,752); N visits (events) testing: 251,759 (832))						
OP	0.841	$3.2x10^{-3}$	0.103	0.165	0.993	0.075
	(0.826, 0.856)	($3.0x10^{-3}$, $3.5x10^{-3}$)	(0.088, 0.120)	(0.140, 0.190)	(0.993, 0.994)	(0.064, 0.088)
LR	0.827	$3.2x10^{-3}$	0.102	0.189	0.992	0.070
	(0.812, 0.842)	($3.0x10^{-3}$, $3.5x10^{-3}$)	(0.087, 0.117)	(0.162, 0.216)	(0.991, 0.992)	(0.060, 0.081)
RF	0.835	$3.2x10^{-3}$	0.095	0.201	0.990	0.062
	(0.820, 0.849)	($3.0x10^{-3}$, $3.4x10^{-3}$)	(0.082, 0.108)	(0.174, 0.228)	(0.990, 0.990)	(0.053, 0.071)
ANN	0.835	$3.2x10^{-3}$	0.111	0.204	0.992	0.076
	(0.821, 0.849)	($3.0x10^{-3}$, $3.4x10^{-3}$)	(0.096, 0.127)	(0.177, 0.233)	(0.991, 0.992)	(0.066, 0.088)
Ensemble:	0.843	$3.2x10^{-3}$	0.105	0.202	0.991	0.071
LR/RF	(0.829, 0.857)	($3.0x10^{-3}$, $3.4x10^{-3}$)	(0.091, 0.120)	(0.175, 0.229)	(0.991, 0.992)	(0.061, 0.082)
Ensemble:	0.842	$3.2x10^{-3}$	0.101	0.200	0.991	0.068
RF/ANN	(0.827, 0.856)	($3.0x10^{-3}$, $3.4x10^{-3}$)	(0.087, 0.116)	(0.173, 0.227)	(0.991, 0.991)	(0.058, 0.078)
Ensemble:	0.834	$3.2x10^{-3}$	0.108	0.198	0.992	0.074
LR/ANN	(0.819, 0.849)	($3.0x10^{-3}$, $3.4x10^{-3}$)	(0.093, 0.123)	(0.171, 0.227)	(0.991, 0.992)	(0.063, 0.085)
Ensemble:	0.843	$3.2x10^{-3}$	0.107	0.201	0.992	0.073
LR/RF/ANN	(0.829, 0.857)	($3.0x10^{-3}$, $3.4x10^{-3}$)	(0.092, 0.122)	(0.174, 0.228)	(0.991, 0.992)	(0.063, 0.084)

Supplementary Table 10 continued. Prediction performance in validation data for **suicide attempt in the 90 days** following an outpatient general medical visit with a mental health diagnosis across race and ethnicity; 95% confidence intervals constructed using 10,000 bootstrap samples. AUC = Area under the receiver operating curve; PPV = Positive predicted value; OP: original parsimonious; LR=Logistic regression with Lasso variable selection; RF = random forest; ANN = artificial neural network

Prediction Model	AUC (95% CI)	Brier score (95% CI)	F-score, 99th percentile (95% CI)	Sensitivity, 99th percentile (95% CI)	Specificity, 99th percentile (95% CI)	PPV, 99th percentile (95% CI)
Native Hawaiian / Pacific Islander (N visits (events) training: 66,571 (174); N visits (events) testing: 29,064 (96))						
OP	0.871	$3.2x10^{-3}$	0.115	0.229	0.991	0.077
	(0.830, 0.909)	($2.6x10^{-3}$, $3.9x10^{-3}$)	(0.073, 0.160)	(0.148, 0.317)	(0.990, 0.992)	(0.048, 0.109)
LR	0.868	$3.2x10^{-3}$	0.132	0.260	0.991	0.088
	(0.826, 0.907)	($2.6x10^{-3}$, $3.8x10^{-3}$)	(0.087, 0.179)	(0.174, 0.351)	(0.990, 0.992)	(0.057, 0.122)
RF	0.854	$3.2x10^{-3}$	0.108	0.219	0.991	0.072
	(0.808, 0.896)	($2.6x10^{-3}$, $3.8x10^{-3}$)	(0.067, 0.153)	(0.138, 0.307)	(0.989, 0.992)	(0.043, 0.103)
ANN	0.873	$3.2x10^{-3}$	0.124	0.240	0.991	0.083
	(0.831, 0.910)	($2.6x10^{-3}$, $3.8x10^{-3}$)	(0.079, 0.171)	(0.156, 0.330)	(0.990, 0.992)	(0.052, 0.117)
Ensemble:	0.867	$3.2x10^{-3}$	0.133	0.260	0.991	0.090
LR/RF	(0.823, 0.907)	($2.6x10^{-3}$, $3.8x10^{-3}$)	(0.087, 0.181)	(0.174, 0.352)	(0.990, 0.992)	(0.057, 0.124)
Ensemble:	0.867	$3.2x10^{-3}$	0.127	0.250	0.991	0.085
RF/ANN	(0.823, 0.907)	($2.6x10^{-3}$, $3.8x10^{-3}$)	(0.082, 0.173)	(0.165, 0.341)	(0.990, 0.992)	(0.054, 0.119)
Ensemble:	0.873	$3.2x10^{-3}$	0.128	0.250	0.991	0.086
LR/ANN	(0.832, 0.911)	($2.6x10^{-3}$, $3.8x10^{-3}$)	(0.083, 0.175)	(0.167, 0.341)	(0.990, 0.992)	(0.055, 0.120)
Ensemble:	0.870	$3.2x10^{-3}$	0.124	0.240	0.991	0.084
LR/RF/ANN	(0.827, 0.910)	($2.6x10^{-3}$, $3.8x10^{-3}$)	(0.079, 0.170)	(0.155, 0.330)	(0.990, 0.992)	(0.052, 0.118)
Non-Hispanic white (N visits (events) training: 4,521,388 (16,072); N visits (events) testing: 1,918,078 (6,940))						
OP	0.837	$3.5x10^{-3}$	0.110	0.235	0.989	0.072
	(0.832, 0.843)	($3.4x10^{-3}$, $3.6x10^{-3}$)	(0.105, 0.115)	(0.225, 0.245)	(0.989, 0.989)	(0.068, 0.075)
LR	0.843	$3.5x10^{-3}$	0.107	0.229	0.989	0.070
	(0.838, 0.848)	($3.4x10^{-3}$, $3.6x10^{-3}$)	(0.102, 0.112)	(0.219, 0.239)	(0.989, 0.989)	(0.066, 0.073)
RF	0.843	$3.5x10^{-3}$	0.100	0.217	0.989	0.065
	(0.838, 0.848)	($3.4x10^{-3}$, $3.6x10^{-3}$)	(0.095, 0.105)	(0.208, 0.227)	(0.989, 0.989)	(0.062, 0.068)
ANN	0.841	$3.5x10^{-3}$	0.113	0.240	0.989	0.074
	(0.836, 0.846)	($3.4x10^{-3}$, $3.6x10^{-3}$)	(0.108, 0.118)	(0.231, 0.251)	(0.989, 0.989)	(0.070, 0.077)
Ensemble:	0.849	$3.5x10^{-3}$	0.109	0.236	0.989	0.071
LR/RF	(0.844, 0.854)	($3.4x10^{-3}$, $3.6x10^{-3}$)	(0.104, 0.114)	(0.226, 0.246)	(0.989, 0.989)	(0.068, 0.074)
Ensemble:	0.848	$3.5x10^{-3}$	0.112	0.243	0.989	0.073
RF/ANN	(0.843, 0.853)	($3.4x10^{-3}$, $3.6x10^{-3}$)	(0.107, 0.117)	(0.233, 0.253)	(0.989, 0.989)	(0.070, 0.076)
Ensemble:	0.844	$3.5x10^{-3}$	0.111	0.240	0.989	0.072
LR/ANN	(0.838, 0.849)	($3.4x10^{-3}$, $3.6x10^{-3}$)	(0.106, 0.116)	(0.230, 0.250)	(0.989, 0.989)	(0.069, 0.076)
Ensemble:	0.848	$3.5x10^{-3}$	0.113	0.243	0.989	0.074
LR/RF/ANN	(0.843, 0.853)	($3.4x10^{-3}$, $3.6x10^{-3}$)	(0.108, 0.118)	(0.233, 0.253)	(0.989, 0.989)	(0.070, 0.077)
Hispanic (N visits (events) training: 1,600,730 (4,339); N visits (events) testing: 682,587 (1,863))						
OP	0.843	$2.7x10^{-3}$	0.091	0.166	0.993	0.063
	(0.833, 0.853)	($2.6x10^{-3}$, $2.8x10^{-3}$)	(0.082, 0.101)	(0.149, 0.183)	(0.993, 0.993)	(0.056, 0.070)
LR	0.839	$2.7x10^{-3}$	0.095	0.177	0.993	0.065
	(0.829, 0.849)	($2.6x10^{-3}$, $2.8x10^{-3}$)	(0.085, 0.104)	(0.160, 0.194)	(0.993, 0.993)	(0.058, 0.072)
RF	0.833	$2.7x10^{-3}$	0.092	0.192	0.992	0.061
	(0.823, 0.843)	($2.6x10^{-3}$, $2.8x10^{-3}$)	(0.084, 0.101)	(0.174, 0.210)	(0.992, 0.992)	(0.055, 0.067)
ANN	0.839	$2.7x10^{-3}$	0.100	0.184	0.993	0.069
	(0.829, 0.849)	($2.6x10^{-3}$, $2.8x10^{-3}$)	(0.091, 0.110)	(0.166, 0.202)	(0.993, 0.993)	(0.062, 0.076)
Ensemble:	0.843	$2.7x10^{-3}$	0.098	0.193	0.992	0.065
LR/RF	(0.833, 0.853)	($2.6x10^{-3}$, $2.8x10^{-3}$)	(0.088, 0.107)	(0.175, 0.211)	(0.992, 0.993)	(0.059, 0.072)
Ensemble:	0.842	$2.7x10^{-3}$	0.101	0.201	0.992	0.067
RF/ANN	(0.832, 0.852)	($2.6x10^{-3}$, $2.8x10^{-3}$)	(0.091, 0.110)	(0.182, 0.219)	(0.992, 0.993)	(0.061, 0.074)
Ensemble:	0.842	$2.7x10^{-3}$	0.097	0.180	0.993	0.066
LR/ANN	(0.832, 0.851)	($2.6x10^{-3}$, $2.8x10^{-3}$)	(0.087, 0.106)	(0.163, 0.198)	(0.993, 0.993)	(0.059, 0.073)
Ensemble:	0.845	$2.7x10^{-3}$	0.098	0.189	0.993	0.066
LR/RF/ANN	(0.835, 0.854)	($2.6x10^{-3}$, $2.8x10^{-3}$)	(0.088, 0.107)	(0.172, 0.208)	(0.992, 0.993)	(0.059, 0.073)

Supplementary Table 10 continued. Prediction performance in validation data for **suicide attempt in the 90 days** following an outpatient general medical visit with a mental health diagnosis across race and ethnicity; 95% confidence intervals constructed using 10,000 bootstrap samples. AUC = Area under the receiver operating curve; PPV = Positive predicted value; OP: original parsimonious; LR=Logistic regression with Lasso variable selection; RF = random forest; ANN = artificial neural network

Prediction Model	AUC (95% CI)	Brier score (95% CI)	F-score, 99th percentile (95% CI)	Sensitivity, 99th percentile (95% CI)	Specificity, 99th percentile (95% CI)	PPV, 99th percentile (95% CI)
Race Unknown (N visits (events) training: 220,416 (772); N visits (events) testing: 93,637 (273))						
OP	0.832	$2.8x10^{-3}$	0.094	0.172	0.993	0.064
	(0.803, 0.860)	($2.5x10^{-3}$, $3.2x10^{-3}$)	(0.069, 0.120)	(0.129, 0.219)	(0.992, 0.993)	(0.047, 0.083)
LR	0.801	$2.8x10^{-3}$	0.090	0.143	0.994	0.066
	(0.770, 0.832)	($2.5x10^{-3}$, $3.2x10^{-3}$)	(0.065, 0.117)	(0.103, 0.186)	(0.994, 0.995)	(0.047, 0.087)
RF	0.816	$2.8x10^{-3}$	0.179	0.289	0.994	0.129
	(0.787, 0.844)	($2.5x10^{-3}$, $3.2x10^{-3}$)	(0.144, 0.212)	(0.235, 0.343)	(0.994, 0.995)	(0.103, 0.156)
ANN	0.813	$2.8x10^{-3}$	0.097	0.150	0.994	0.072
	(0.784, 0.842)	($2.5x10^{-3}$, $3.2x10^{-3}$)	(0.071, 0.126)	(0.109, 0.193)	(0.994, 0.995)	(0.052, 0.094)
Ensemble:	0.821	$2.8x10^{-3}$	0.132	0.212	0.994	0.096
LR/RF	(0.792, 0.850)	($2.5x10^{-3}$, $3.2x10^{-3}$)	(0.102, 0.163)	(0.165, 0.263)	(0.994, 0.995)	(0.073, 0.120)
Ensemble:	0.825	$2.8x10^{-3}$	0.167	0.275	0.994	0.120
RF/ANN	(0.797, 0.853)	($2.5x10^{-3}$, $3.2x10^{-3}$)	(0.134, 0.200)	(0.223, 0.329)	(0.994, 0.995)	(0.095, 0.146)
Ensemble:	0.810	$2.8x10^{-3}$	0.097	0.154	0.994	0.071
LR/ANN	(0.780, 0.840)	($2.5x10^{-3}$, $3.2x10^{-3}$)	(0.071, 0.125)	(0.112, 0.198)	(0.994, 0.995)	(0.051, 0.092)
Ensemble:	0.823	$2.8x10^{-3}$	0.104	0.161	0.994	0.077
LR/RF/ANN	(0.794, 0.851)	($2.5x10^{-3}$, $3.2x10^{-3}$)	(0.076, 0.133)	(0.119, 0.205)	(0.994, 0.995)	(0.056, 0.099)

Supplementary Table 11. Prediction performance in validation data for **suicide attempt in the 90 days** following an outpatient general medical visit with a mental health diagnosis across sex; 95% confidence intervals constructed using 10,000 bootstrap samples. AUC = Area under the receiver operating curve; PPV = Positive predicted value; OP: original parsimonious; LR=Logistic regression with Lasso variable selection; RF = random forest; ANN = artificial neural network

Prediction Model	AUC (95% CI)	Brier score (95% CI)	F-score, 99th percentile (95% CI)	Sensitivity, 99th percentile (95% CI)	Specificity, 99th percentile (95% CI)	PPV, 99th percentile (95% CI)
Female (N visits (events) training: 4,658,689 (14,554); N visits (events) testing: 1,981,603 (6,598))						
OP	0.850	$3.3x10^{-3}$	0.113	0.222	0.991	0.076
	(0.844, 0.855)	($3.2x10^{-3}$, $3.3x10^{-3}$)	(0.108, 0.118)	(0.212, 0.232)	(0.991, 0.991)	(0.072, 0.079)
LR	0.851	$3.2x10^{-3}$	0.113	0.227	0.991	0.075
	(0.846, 0.856)	($3.2x10^{-3}$, $3.3x10^{-3}$)	(0.108, 0.118)	(0.217, 0.237)	(0.990, 0.991)	(0.071, 0.079)
RF	0.849	$3.2x10^{-3}$	0.103	0.217	0.990	0.068
	(0.845, 0.854)	($3.2x10^{-3}$, $3.3x10^{-3}$)	(0.099, 0.108)	(0.208, 0.227)	(0.990, 0.990)	(0.065, 0.071)
ANN	0.852	$3.2x10^{-3}$	0.118	0.233	0.991	0.079
	(0.847, 0.857)	($3.2x10^{-3}$, $3.3x10^{-3}$)	(0.113, 0.124)	(0.223, 0.243)	(0.991, 0.991)	(0.076, 0.083)
Ensemble:	0.856	$3.2x10^{-3}$	0.115	0.236	0.990	0.076
LR/RF	(0.851, 0.861)	($3.1x10^{-3}$, $3.3x10^{-3}$)	(0.110, 0.120)	(0.226, 0.246)	(0.990, 0.991)	(0.072, 0.080)
Ensemble:	0.856	$3.2x10^{-3}$	0.116	0.237	0.990	0.077
RF/ANN	(0.851, 0.861)	($3.2x10^{-3}$, $3.3x10^{-3}$)	(0.111, 0.121)	(0.227, 0.247)	(0.990, 0.991)	(0.073, 0.080)
Ensemble:	0.853	$3.2x10^{-3}$	0.117	0.232	0.991	0.078
LR/ANN	(0.848, 0.858)	($3.2x10^{-3}$, $3.3x10^{-3}$)	(0.111, 0.122)	(0.223, 0.243)	(0.991, 0.991)	(0.074, 0.082)
Ensemble:	0.857	$3.2x10^{-3}$	0.117	0.237	0.991	0.078
LR/RF/ANN	(0.852, 0.862)	($3.1x10^{-3}$, $3.3x10^{-3}$)	(0.112, 0.123)	(0.227, 0.247)	(0.991, 0.991)	(0.074, 0.082)
Male (N visits (events) training: 2,740,517 (9,651); N visits (events) testing: 1,170,320 (3,961))						
OP	0.819	$3.3x10^{-3}$	0.093	0.200	0.989	0.061
	(0.811, 0.826)	($3.2x10^{-3}$, $3.4x10^{-3}$)	(0.087, 0.099)	(0.188, 0.213)	(0.989, 0.990)	(0.057, 0.065)
LR	0.818	$3.3x10^{-3}$	0.089	0.185	0.990	0.058
	(0.811, 0.825)	($3.2x10^{-3}$, $3.4x10^{-3}$)	(0.083, 0.095)	(0.173, 0.198)	(0.990, 0.990)	(0.054, 0.063)
RF	0.824	$3.3x10^{-3}$	0.092	0.199	0.989	0.060
	(0.816, 0.831)	($3.2x10^{-3}$, $3.4x10^{-3}$)	(0.086, 0.098)	(0.187, 0.211)	(0.989, 0.990)	(0.056, 0.064)
ANN	0.819	$3.3x10^{-3}$	0.096	0.206	0.990	0.063
	(0.812, 0.827)	($3.2x10^{-3}$, $3.4x10^{-3}$)	(0.090, 0.102)	(0.193, 0.218)	(0.989, 0.990)	(0.059, 0.067)
Ensemble:	0.829	$3.3x10^{-3}$	0.093	0.197	0.990	0.060
LR/RF	(0.822, 0.836)	($3.2x10^{-3}$, $3.4x10^{-3}$)	(0.087, 0.099)	(0.185, 0.210)	(0.989, 0.990)	(0.056, 0.065)
Ensemble:	0.828	$3.3x10^{-3}$	0.101	0.220	0.989	0.065
RF/ANN	(0.820, 0.835)	($3.2x10^{-3}$, $3.4x10^{-3}$)	(0.095, 0.107)	(0.207, 0.233)	(0.989, 0.990)	(0.061, 0.070)
Ensemble:	0.821	$3.3x10^{-3}$	0.093	0.201	0.989	0.061
LR/ANN	(0.814, 0.829)	($3.2x10^{-3}$, $3.4x10^{-3}$)	(0.088, 0.099)	(0.189, 0.214)	(0.989, 0.990)	(0.057, 0.065)
Ensemble:	0.829	$3.3x10^{-3}$	0.095	0.204	0.990	0.062
LR/RF/ANN	(0.821, 0.836)	($3.2x10^{-3}$, $3.4x10^{-3}$)	(0.089, 0.101)	(0.191, 0.216)	(0.989, 0.990)	(0.058, 0.066)
