# Supplementary material for: Complex modeling with detailed temporal predictors does not improve health records-based suicide risk prediction
Source: NPJ Digit Med. 2023 Mar 23;6:47. doi: 10.1038/s41746-023-00772-4 (PMC10036475; doi:10.1038/s41746-023-00772-4)
Supplement: Supplementary file 2 — REPORTING SUMMARY [file 41746_2023_772_MOESM2_ESM.pdf]

nature portfolio

Corresponding author(s): Susan M Shortreed

Last updated by author(s): Nov 8, 2022

Reporting Summary

Nature Portfolio wishes to improve the reproducibility of the work that we publish. This form provides structure for consistency and transparency in reporting. For further information on Nature Portfolio policies, see our Editorial Policies and the Editorial Policy Checklist.

Statistics

For all statistical analyses, confirm that the following items are present in the figure legend, table legend, main text, or Methods section.

n/a	Confirmed	
☐	☒	The exact sample size (*n*) for each experimental group/condition, given as a discrete number and unit of measurement
☒	☐	A statement on whether measurements were taken from distinct samples or whether the same sample was measured repeatedly
☒	☐	The statistical test(s) used AND whether they are one- or two-sided<br>*Only common tests should be described solely by name; describe more complex techniques in the Methods section.*
☐	☒	A description of all covariates tested
☒	☐	A description of any assumptions or corrections, such as tests of normality and adjustment for multiple comparisons
☐	☒	A full description of the statistical parameters including central tendency (e.g. means) or other basic estimates (e.g. regression coefficient) AND variation (e.g. standard deviation) or associated estimates of uncertainty (e.g. confidence intervals)
☒	☐	For null hypothesis testing, the test statistic (e.g. *F*, *t*, *r*) with confidence intervals, effect sizes, degrees of freedom and *P* value noted<br>*Give P values as exact values whenever suitable.*
☒	☐	For Bayesian analysis, information on the choice of priors and Markov chain Monte Carlo settings
☒	☐	For hierarchical and complex designs, identification of the appropriate level for tests and full reporting of outcomes
☒	☐	Estimates of effect sizes (e.g. Cohen's *d*, Pearson's *r*), indicating how they were calculated

Our web collection on statistics for biologists contains articles on many of the points above.

Software and code

Policy information about availability of computer code

Data collection	The analytic data set was prepared using SAS software, Version 9 of the SAS System for Windows was used to extract data from vritual data warehouses at each of the health systems. Copyright © 2019 SAS Institute Inc. SAS and all other SAS Institute Inc. product or service names are registered trademarks or trademarks of SAS Institute Inc., Cary, NC, USA.
Data analysis	From the Methods section:Random forests were estimated using R package ranger version 0.11.2, R version 3.5.3 (2019-03-11) and RStudio version 1.1.463. Additional software needed to fit the artificial neural networks included Tensorflow version 2.0, Anaconda version 4.3.30, and Python version 3.6.9. The same software used to implement the artificial neural networks was used to estimate the penalized logistic regression models because of the computational efficiencies and large data capacity available in the Keras package.

For manuscripts utilizing custom algorithms or software that are central to the research but not yet described in published literature, software must be made available to editors and reviewers. We strongly encourage code deposition in a community repository (e.g. GitHub). See the Nature Portfolio guidelines for submitting code & software for further information.

Data

Policy information about availability of data

All manuscripts must include a data availability statement. This statement should provide the following information, where applicable:

- Accession codes, unique identifiers, or web links for publicly available datasets
- A description of any restrictions on data availability
- For clinical datasets or third party data, please ensure that the statement adheres to our policy

The datasets generated and analyzed during this study are not publicly available because they contain detailed information from the electronic health records in the health systems participating in this study and are governed by Health Insurance Portability and Accountability Act (HIPAA). Data are, however, available from the authors upon reasonable request, with permission of all health systems involved and a fully executed data use agreement.

Human research participants

Policy information about studies involving human research participants and Sex and Gender in Research.

Reporting on sex and gender	From Methods: Information on sex represents sex assigned at birth and was extracted from health systems records, which, at the time data were extracted, did not distinguish between sex at birth and gender identity.<br>From limitations (in discussion): Information on gender identity and sexual orientation were not available. At the time of data extraction only sex assigned at birth was available in the health systems records data; all health systems are currently expanding collection of patients' sexual orientation and gender identity, including gender transitions, which could improve model performance overall and among sexual and gender minorities, populations for which suicide prevention research is critical.
Population characteristics	First two paragraphs of the results section, Table 1, and Supplementary Table 1 describe the sample.
Recruitment	N/A
Ethics oversight	From Methods:<br>Responsible institutional review boards for each participating health system approved waivers of consent for use of records data in this research: Henry Ford Health institutional review board ([IRB], #9998, Henry Ford Health System), Kaiser Permanente Colorado IRB (#00002931, Kaiser Permanente Colorado), and Kaiser Permanente Interregional IRB (#799744, Washington, HealthPartners, Hawaii, Northwest, and Southern California regions of Kaiser Permanente).

Note that full information on the approval of the study protocol must also be provided in the manuscript.

Field-specific reporting

Please select the one below that is the best fit for your research. If you are not sure, read the appropriate sections before making your selection.

☐ Life sciences ☒ Behavioural & social sciences ☐ Ecological, evolutionary & environmental sciences

For a reference copy of the document with all sections, see nature.com/documents/nr-reporting-summary-flat.pdf

Behavioural & social sciences study design

All studies must disclose on these points even when the disclosure is negative.

Study description	Quantitative research to build risk prediction models for suicide attempt using data from health records and insurance claims.
Research sample	From Methods: All outpatient mental health visits made by individuals 11 years and older between January 1, 2009 and September 30, 2017 in seven health care systems (HealthPartners, Henry Ford Health System, and the Colorado, Hawaii, Northwest, Southern California, and Washington regions of Kaiser Permanente) were included. An outpatient mental health visit was defined as an outpatient visit to a mental health specialty provider or a visit, with a mental health diagnosis, made to a general medical provider (referred to here as general medical visits).
Sampling strategy	N/A - all eligible visits were included.
Data collection	Data were extracted from health records and insurance claims
Timing	January 1, 2009 and September 30, 2017

Data exclusions	None
Non-participation	N/A
Randomization	N/A

Reporting for specific materials, systems and methods

We require information from authors about some types of materials, experimental systems and methods used in many studies. Here, indicate whether each material, system or method listed is relevant to your study. If you are not sure if a list item applies to your research, read the appropriate section before selecting a response.

Materials & experimental systems

n/a	Involved in the study	
☒	☐	Antibodies
☒	☐	Eukaryotic cell lines
☒	☐	Palaeontology and archaeology
☒	☐	Animals and other organisms
☐	☒	Clinical data
☒	☐	Dual use research of concern

Methods

n/a	Involved in the study	
☒	☐	ChIP-seq
☒	☐	Flow cytometry
☒	☐	MRI-based neuroimaging

Clinical data

Policy information about clinical studies

All manuscripts should comply with the ICMJE guidelines for publication of clinical research and a completed CONSORT checklist must be included with all submissions.

Clinical trial registration	N/A
Study protocol	N/A
Data collection	From Methods: All outpatient mental health visits made by individuals 11 years and older between January 1, 2009 and September 30, 2017 in seven health care systems (HealthPartners, Henry Ford Health System, and the Colorado, Hawaii, Northwest, Southern California, and Washington regions of Kaiser Permanente) were included. An outpatient mental health visit was defined as an outpatient visit to a mental health specialty provider or a visit, with a mental health diagnosis, made to a general medical provider (referred to here as general medical visits).
Outcomes	From Methods: Suicides were identified using state death certificates with cause of death ICD-10 mortality codes in the ranges X60-X84, Y10-Y34, Y87.0, and Y87.2 Nonfatal suicide attempts were identified using diagnosis codes from electronic health records and insurance claims. Non-fatal suicide attempts on or before September 30, 2015 were identified using ICD-9-CM diagnosis codes E950-E958 (suicide and self-inflicted injury) or E980-E988 (injury of undetermined intent). After September 30, 2015, non-fatal suicide attempts were identified using ICD-10-CM diagnosis codes were used to identify non-fatal suicide attempts. The full list of ICD-10-CM diagnosis codes used to identify non-fatal suicide attempts includes over 1000 (non-adjacent) codes and can be found at: https://github.com/MHResearchNetwork/more-srpm. We briefly summarize here. An ICD-10 era attempt was defined as either:1) the presence of any single code from the following ranges: a) X71-X83 (external causes of morbidity classified as intentional self-harm), b) Y21-Y33 (external causes of morbidity of undetermined intent), c) T36-T65 (poisoning/toxic effects) or T71 (asphyxiation) initial encounter codes with “intentional self-harm” or “undetermined intent” in the official code description, or d) T14.91 (suicide attempt); or 2) the presence of suicidal ideation code R45.851 accompanied by an initial encounter code for a wound (S/T codes with “wound,” “laceration,” or “traumatic amputation” in the description) or poisoning/toxic effects (T codes with “poisoning” or “toxic” in the description) recorded in the same encounter.
